# DIMORPH: an integrated multi-omics resource for camptothecin-producing plants

**DOI:** 10.1186/s43897-025-00225-4

**Published:** 2026-06-03

**Authors:** Qian Lou, Xiangdong Pu, Wenjie Xu, Ranran Gao, Longlong Gao, Zhichao Xu, Zhe Wang, Xinyao Li, Tianyi Xin, Haitao Li, Wu Wang, Deying Tang, Anshun Xu, Guihong Qi, Yutong Gan, Jinlan Zhang, Jingyuan Song

**Affiliations:** 1 State Key Laboratory of Bioactive Substance and Function of Natural Medicines, Institute of Medicinal Plant Development, Chinese Academy of Medical Sciences & Peking Union Medical College, Beijing, 100193 China; 2 Anhui Provincial Laboratory of Inflammatory and Immunity Disease, Anhui Institute of Innovative Drugs, School of Pharmacy, Hefei, 230032 China; 3 Key Laboratory of Beijing for Identification and Safety Evaluation of Chinese Medicine, China Academy of Chinese Medical Sciences, Institute of Chinese Materia Medica, Beijing, 100700 China; 4 College of Life Science, Northeast Forestry University, Harbin, 150040 China; 5 State Key Laboratory of Bioactive Substances and Functions of Natural Medicines, Institute of Materia Medica, Chinese Academy of Medical Sciences and Peking Union Medical College, Beijing, 100050 China; 6 Yunnan Key Laboratory of Southern Medicinal Resources, Yunnan Branch Institute of Medicinal Plant Development, Chinese Academy of Medical Sciences, Jinghong, 666100 China; 7 Key Laboratory of Chinese Medicine Resources Conservation, State Administration of Traditional Chinese Medicine of the People’s Republic of China, Engineering Research Center of Chinese Medicine Resource, Ministry of Education, Beijing, 100193 China

**Keywords:** Omics database, Camptothecin, Camptothecin hydroxylase, Regio-selectivity, High-efficiency mutant

## Abstract

Over the past decade, extensive multi-omics data of camptothecin (CPT)-producing plants have been generated, resulting in a wealth of genomic resources. However, to date, no available database supports utilization for understanding biological mechanisms of CPT biosynthesis. In this study, we constructed DIMORPH (https://www.dimorph.cn:9000.), a Database Integrated Multiple Omics Resources for CPT-Producing Herbs. The database consolidated genomic, transcriptomic, and metabolic data of the three representative CPT-producing plants including *Camptotheca acuminata*, *Ophiorrhiza pumila* and *Nothapodytes nimmoniana*, and integrated functional annotation, synteny, and co-expression analytical results. Utilizing DIMORPH, a new CPT 10-hydroxylase (CPT10H), CYP81BQ24, was identified in *C. acuminata*. Structural comparison between CYP81BQ24 and CYP81BQ23 (CPT 11 hydroxylase), combined with fragment substitution mutagenesis, revealed ten key amino acid residues determined the regio-selectivity of CPT hydroxylases (CPTHs) at C-10 and C-11 position. Additionally, a high-efficiency CPT10H mutant was obtained through site-directed mutagenesis guided by sequence alignment and structural prediction of 17 CYP450 hydroxylases. Therefore, the comprehensive database focused on CPT will be helpful to the elucidation of CPT biosynthetic pathway and production of CPT-derived drugs.

**Supplementary Information:**

The online version contains supplementary material available at 10.1186/s43897-025-00225-4.

## Core

This study constructed a database integrated multi-omics resources for CPT-producing plants. Based on the database, a new CPT 10-hydroxylase (CPT10H), CYP81BQ24, was identified in *C. acuminata*. Mutagenesis results revealed ten key residues determined the regio-selectivity of CPT hydroxylases (CPTHs) at C-10 and C-11 position and obtained a high-efficiency CPT10H mutant. The comprehensive database focused on CPT will be helpful to the elucidation of CPT biosynthetic pathway and production of CPT-derived drugs.

## Gene & accession numbers

Sequencing data were deposited under the National Genomics Data Center accession numbers PRJCA028313, PRJCA028705 and PRJCA028323.

## Introduction

Camptothecin (CPT) is a valuable natural product for inhibitory activity against topoisomerase I. CPT-derived drugs, such as irinotecan and topotecan, are widely utilized as first-line therapies for various cancers, generating an annual market value exceeding 10 billion yuan (Bailly 2019; Ormrod et al. 1999). However, the endogenous content of CPT in plants is extremely low, and its chemical synthesis requires the use of organic reagents (Ramesha et al. 2013; Zhao et al. 2011). In recent years, elucidating the biosynthetic pathway of CPT and developing heterologous production systems have emerged as key strategies to overcome these production challenges.

**Fig. 1:**
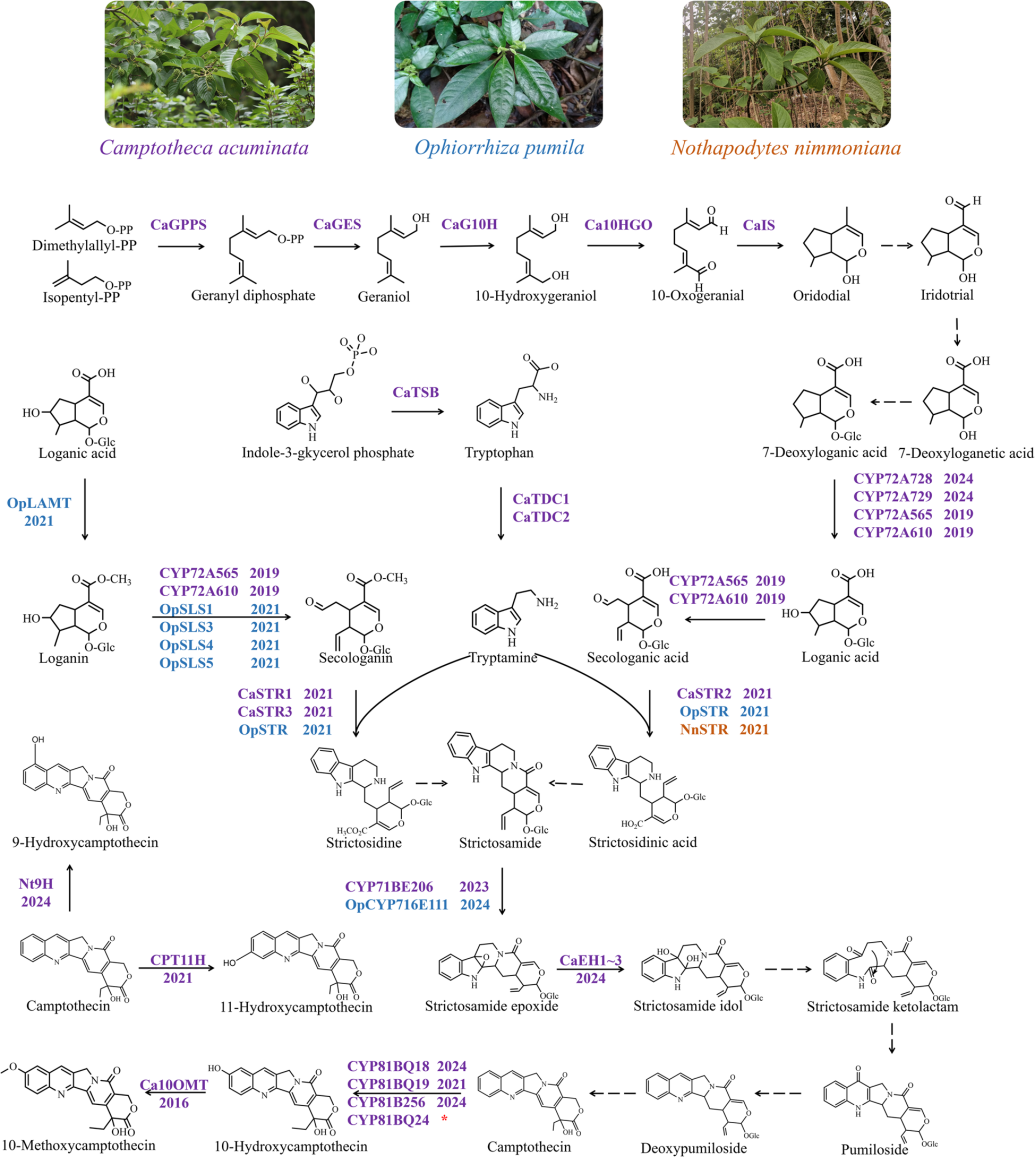
Advances in biosynthetic pathways of camptothecin. Purple: Validated enzymes in *Camptotheca acuminata*, Blue: Validated enzymes in *Ophiorrhiza pumila*, Orange: Validated enzymes in *Nothapodytes nimmoniana*. Solid arrow: Deciphered steps; Dotted arrow: Undeciphered steps

Multiple omics datasets have significantly advanced research on the biosynthesis of valuable natural products (Pu et al. 2020; Xu et al. 2022, 2024, 2020). In recent years, genomic (Kang et al. 2021; Rai et al. 2021), transcriptomic (Rather et al. 2018; Yamazaki et al. 2013; Zhao et al. 2017), and metabolomic (Pu et al. 2019; Sadre et al. 2016) data of CPT-producing species have been systematically investigated, with a particular focus on three representative plants: *Camptotheca acuminata*, *Ophiorrhiza pumila*, and *Nothapodytes nimmoniana*. These multi-omics datasets have facilitated the identification of several key enzymes in the CPT biosynthetic pathway (Fan et al. 2022) (Fig. 1). However, to date, no database has been established to comprehensively integrate these datasets. Moreover, although several CPT 10-hydroxylases have been reported (Nguyen et al. 2021; Pu et al. 2024), the underlying mechanisms governing the regio-selectivity remain unknown, and no attempts have been made to construct a high-efficiency mutant.

In this study, we developed DIMORPH (Database Integrated Multiple Omics Resources for CPT-Producing Herbs, http://dimorph.cn:9000/), a dedicated database centered on CPT. By integrating publicly available and newly generated genomic, transcriptomic, and metabolomic data from the three representative CPT-producing species, DIMORPH provides a comprehensive resource for CPT-related research. Through in-depth mining of DIMORPH, we identified and characterized a novel CPT 10-hydroxylase (CPT10H), CYP81BQ24, from *C. acuminata*. Further comparative analysis of CYP81BQ24 and CYP81BQ23 (CPT 11-hydroxylase, CPT11H) revealed key sites that determine the regio-selectivity of CPT hydroxylases (CPTHs). Based on these findings, mutations were introduced into CPT10H, resulting in a highly efficient mutant. This study not only provided a valuable platform for CPT-related research but also laid a foundation for understanding the CPT biosynthesis pathway and advancing synthetic biology research on CPT-derived drugs.

## Results

### Construction of a database integrated multiple omics resources for CPT-producing herbs (DIMORPH)

In total, four genomes, transcriptome data of 48 samples and metabolome data of 66 samples from *C. acuminata*, *O. pumila*, and *N. nimmoniana* were integrated. These data were derived from two parts (Table 1). The genome of *N. nimmoniana* (raw illumina reads), transcriptome of 18 samples from *C. acuminata* and nine samples from *O. pumila*, and metabolome of 36 samples from *C. acuminata* were newly generated for this paper. Three genomes of *C. acuminata* and *O. pumila*, transcriptomes of 19 samples from *C. acuminata* and two samples from *N. nimmoniana*, and metabolomic data of 30 samples from *O. pumila* were downloaded from published researches. All the data were analyzed and the results were integrated in the database.

**Table 1:** Summary of data in the database integrated multiple omics resources for CPT-producing herbs

	*Camptotheca acuminata*	*Ophiorrhiza pumila*	*Nothapodytes nimmoniana*
Genome	Figshare (Published in 2021) (Kang et al. 2021)	National Center for Biotechnology Information and a dedicated server (http://pumila.kazusa.or.jp/) (Published in 2021) (Rai et al. 2021)	This study (Genome survey)
	Dryad Digital Repository (Published in 2017) (Zhao et al. 2017)	/	/
Transcriptome	Reference (Zhao et al. 2017) This study	This study	Reference (Rather et al. 2018)
Metabolome	Reference (Sadre et al. 2016) This study	Reference (Yamazaki et al. 2013)	References (Namdeo et al. 2012; Wu et al. 2008)

**Fig. 2:**
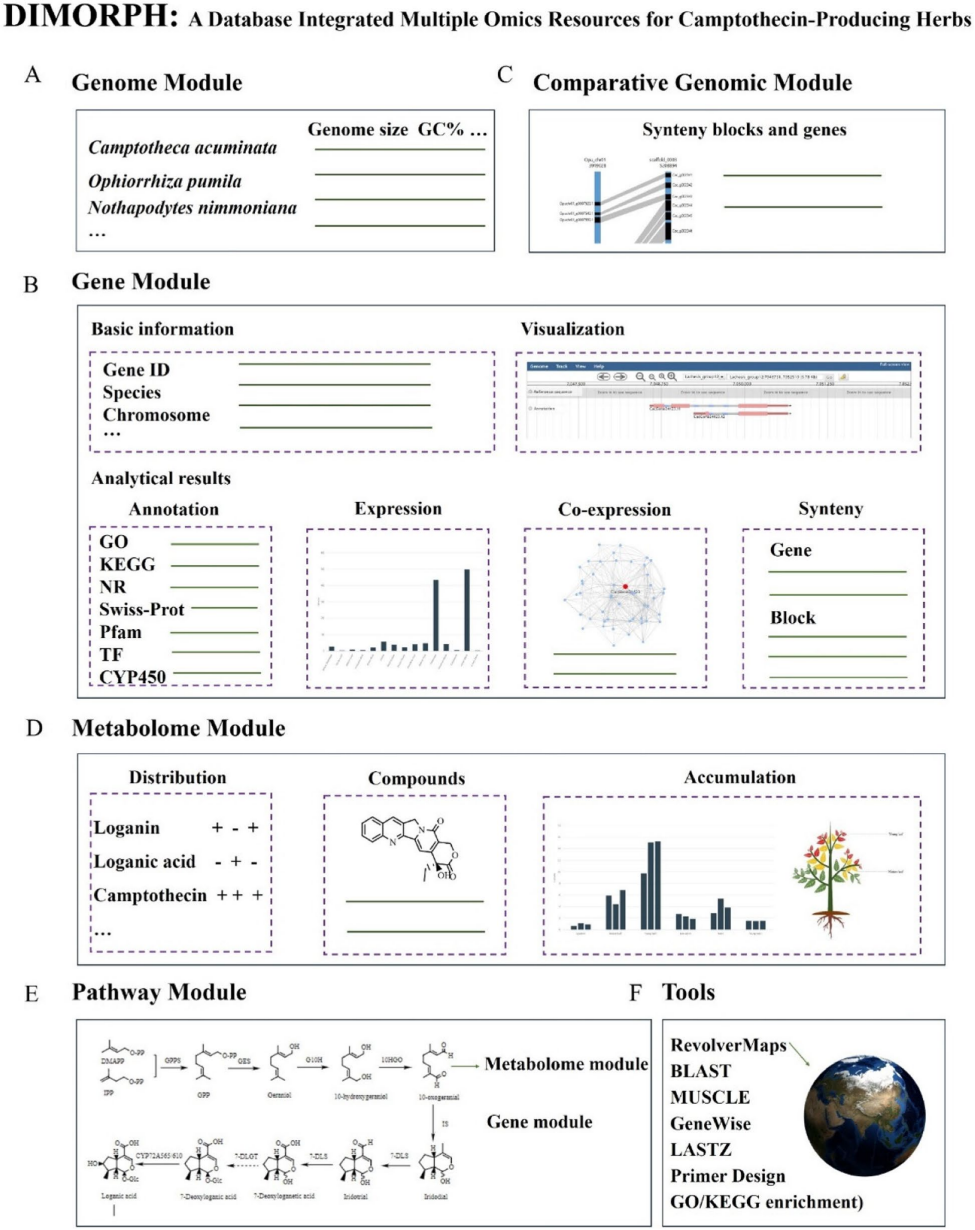
Schematic of the database integrated multiple omics resources for CPT-producing herbs. **A** Genome module. **B** Gene module. **C** Comparative genomics module. **D** Metabolome module. **E** The biosynthetic pathway of camptothecin module. **F** Functional tools module

The current DIMORPH website comprised five modules: (1) genome module, (2) gene module, (3) comparative genomics module, (4) metabolome module, (5) pathway module, and (6) tools (Fig. 2).

The genome module included assembly summary. In total, three assembled genomes of *C. acuminata* and *O. pumila*, and the sequencing data of *N. nimmoniana* were integrated in the database. In the genome module, the basic information of the three genome assemblies including sequencing platform, assembled level, assembly size, GC%, the number of contigs and scaffolds, contig N50, and scaffold N50 was presented in a table for users to quickly understand the quality of each genome stored in the database (Fig. 2A).

The gene module provides detailed information on a gene of interest. This module contains two aspects of information (Fig. 2B). One is the basic information including gene ID, locus, gene length, strand and coding sequences of target ones. The JBrowse results were embedded for visualization. The other contains bioinformatic results including functional annotation, expression level, co-expression network, syntenic blocks and genes. A total of 288,355 functional elements from the three genomes were annotated using five databases (Table S4). DIMORPH identified 342 and 258 putative CYP450 genes in the two genomes of *C. acuminata* and 380 of that in the genome of *O. pumila*. Genome-wide analysis of TFs identified 93, and 94 transcriptional factors families in the two genomes of *C. acuminata*, and 91 in the genome of *O. pumila* (Table S5), covering all major 58 TF families in plants.

The comparative genomics module supported synteny analysis of two genomes. A total of 2,751 synteny blocks and 104,714 collinearity genes were predicted in DIMORPH (Table S6). In the comparative genome module (Fig. 2C), synteny blocks between the selected two genomes are listed in rows. Detailed information of collinear gene pairs is shown in two formats, table and dot-plot diagrams. The gene page is linked by clicking the gene ID. Gene pages also contain information related to synteny blocks and collinear genes.

The metabolome module presented the distribution and accumulation of CPT-derived metabolites. In the metabolome module, key compounds involved in the biosynthetic pathway of CPT that can be detected in each of the three species were first listed in table (Fig. 2D). Detailed page provides compound-related data including chemical structural formula, molecular formula, MS/MS fragments, and pharmacological research. In addition, the accumulation levels of the compound in different tissues of the three species were shown by bar charts, box plots, and plant mode diagrams.

The Pathway module is the page upon which DIMORPH displays all relevant omics data with the overall CPT biosynthetic pathway at its core. Putative biosynthetic pathway of CPT in the three species was mapped (Fig. 2E). The chemical structural formulas were linked with the metabolome interface. Key enzymes which catalyze the reaction between two compounds are labeled upon the arrow, and if the functions of the enzymes were identified, the gene interface and corresponding references were linked. For enzymes those have not yet been reported from *C. acuminata*, related references for homologous genes characterized in *Catharanthus roseus* were provided.

Functional tools were deployed. DIMORPH has provided functional tools and units so that users can perform analysis or statistics on the website itself (Fig. 2F). Four types of tools were embedded in the database which enabled website monitoring (RevolverMaps), sequence alignment (BLAST, MUSCLE, GeneWise and LASTZ), primer design and functional annotation (GO/KEGG enrichment). In particular, RevolverMaps provided the number and IP addresses of the visiting users.

### Discovery and functional identification of CYP81BQ24

**Fig. 3:**
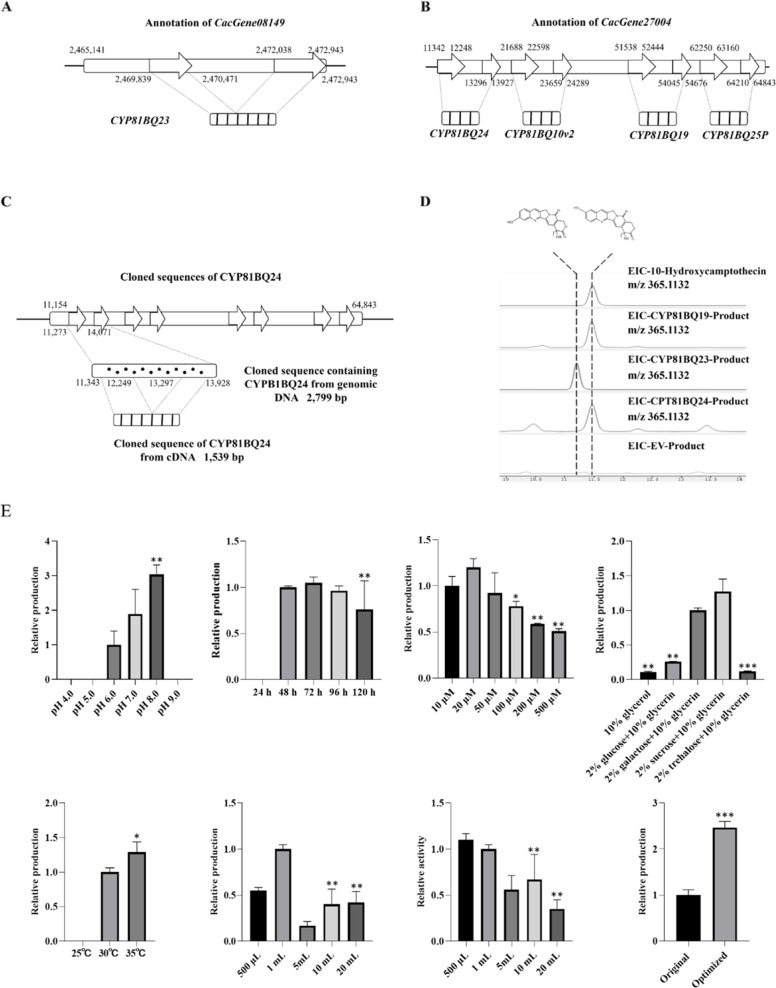
Functional characterization of CYP81BQ24. **A** Annotation of *CacGene08149*. **B** Annotation of *CacGene27004*. **C** Alignment of cloned sequences of *CYP81BQ24*. **D** Extracted ion chromatograms from LC–MS analysis of in vivo reactions catalyzed by CYP81BQ24. **E** Optimization of in vivo catalytic reaction conditions for CYP81BQ24. * *P* < 0.05; ** *P* < 0.01, *** *P* < 0.001; **** *P* < 0.0001

In the pathway module, all the validated enzymes were listed corresponding to genes annotated in the genome. However, alignment results revealed that both *CYP81BQ19* (CPT10H) and *CYP81BQ23* aligned to the regions that were annotated as non-coding sequences (Figure S1). *CYP81BQ23* aligned to the region from 2,469,839 bp to 2,470,476 bp and 2,472,034 bp to 2,472,943 bp of *CacGene08149*, where the region from 2,469,839 bp to 2,470,476 bp was annotated as non-coding sequence (Fig. 3A). *CYP81BQ19* aligned to the region from 51,538 bp to 52,444 bp and 54,045 bp to 54,676 bp of *CacGene27004* (Fig. 3B), where both regions were annotated as non-coding sequences. Using newly generated transcriptome datasets as reference, the regions where *CYP81BQ23* and *CYP81BQ19* aligned to both have a relatively high coverage of reads (Figure S1). Thus, *CacGene08149* and *CacGene27004* were manually annotated. *CacGene27004* was annotated as four genes: *CYP81BQ24*, located from 11,342 to 12,248 bp and 13,296 to 13,927 bp; *CYP81BQ10v2*, located from 21,688 to 22,598 bp and 23,659 to 24,289 bp; *CYP81BQ19*, located from 51,538 to 52,444 bp and 54,045 to 54,676 bp, and *CYP81BQ25P*, located from 62,250 to 63,160 bp and 64,210 to 64,843 bp (Fig. 3B). The coding sequences of *CYP81BQ23*, *CYP81BQ24*, *CYP81BQ10v2*, *CYP81BQ19* and *CYP81BQ25P* were cloned from the cDNA of *C. acuminata*. Cloned sequences were consistent with annotated results except for the termination mutation in the sequence of *CYP81BQ25P.*

Alignment results revealed that CYP81BQ24 exhibited a sequence similarity over 80% with both CYP81BQ19 and CYP81BQ23, and clustered with them in the phylogenetic tree (Figure S2). Thus, CYP81BQ24 was selected as a CPTH candidate. To verify *CYP81BQ24* is a new gene, a sequence of 2,799 bp containing *CYP81BQ24* was cloned from the genomic DNA of *C. acuminata*. The results showed that cloned sequence was consistent with the annotation and different from the region to which *CYP81BQ23* and *CYP81BQ19* were aligned (Fig. 3C). That is, CYP81BQ24 is highly likely to be a new CPTH. Thus, the opening-reading frame of *CYP81BQ24* was cloned to pYES2-Ura vector and both in vivo and in vitro assay of yeast transformed with pYES2-Ura -*CYP81BQ24* showed the formation of a new product with m/z 365.1132. The retention time, ultraviolet absorption peak and characteristic cleavages were consistent with 10-hydroxycamptothecin (10HCPT) (Fig. 3D, S3), which validated CPT-10 hydroxylation catalytic activity of CYP81BQ24. Comparison of CPT10Hs with in vivo catalytic assay revealed that CYP81BQ24 exhibited reduced activity than CYP81BQ19, but demonstrated higher catalytic activity compared to the CPT 10-hydroxylases reported before (CYP81BQ18 and CYP81B256) (Figure S4).

### In vivo reaction condition optimization of CYP81BQ24

In total, the effects of six conditions including reaction time, temperature, pH, volume, carbon sources and substrate concentration on the production of 10HCPT were examined (Fig. 3E). The results showed none of the current conditions were optimal. Compared with them, optimal conditions including pH of medium (8.0), cultivation temperature (35 ℃) and volume (20 mL) had significant influence on the production of 10HCPT, while conditions of reaction time, carbon source and substrate concentration had effect on the production with no significant difference. Adjusting the pH of the medium from 6.0 (standard) to 8.0 (optimum) resulted in nearly triple the yield. Increasing the reaction temperature from 30 ℃ to 35℃ could only increase the yield by 20%. The production of 10HPCT increased as the reaction volume increased, but in reality, the catalytic efficiency was maximum at a reaction volume of 500 μL.

### Comparison of the protein structures of CPTHs

**Fig. 4:**
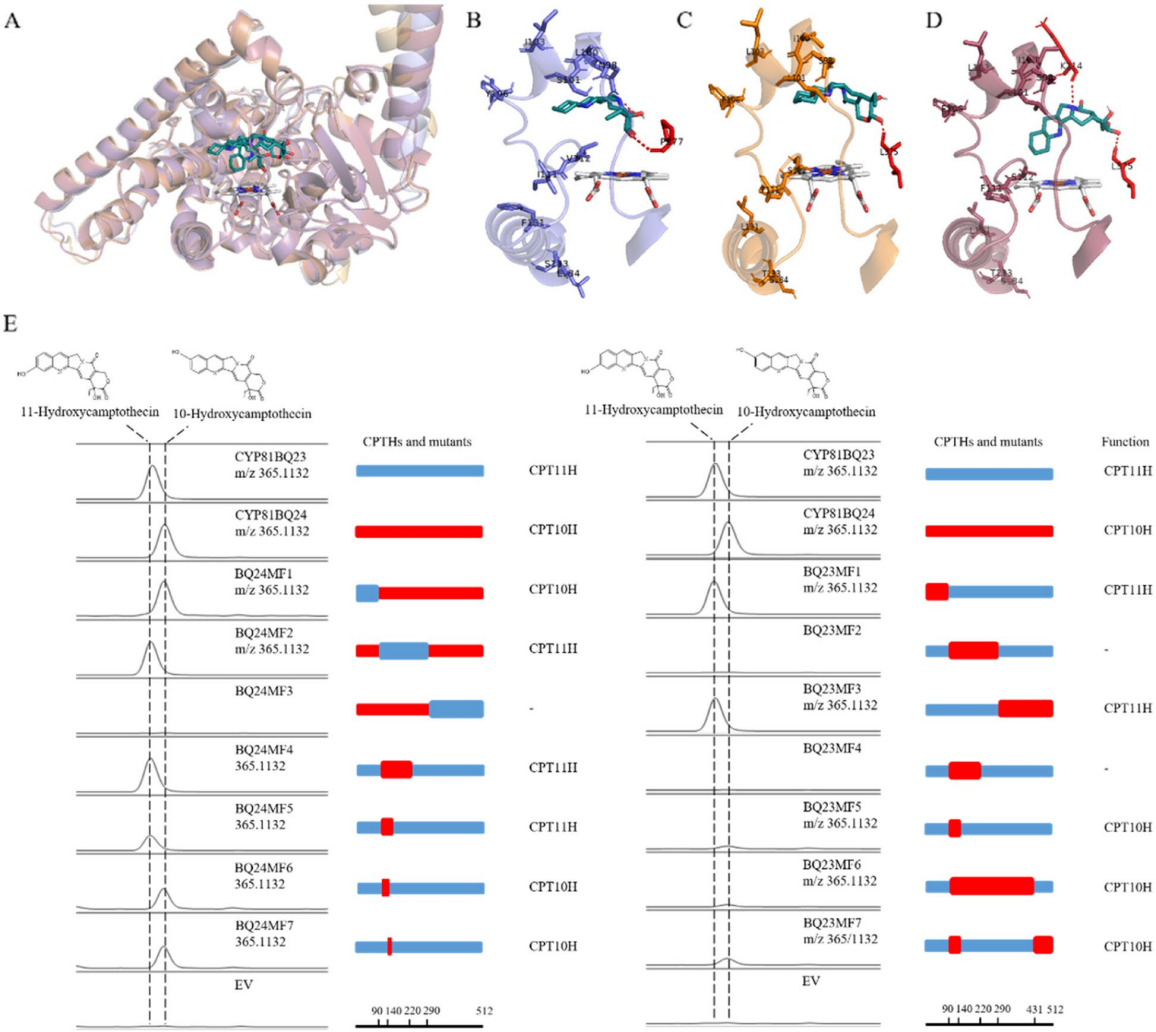
Predicted 3D structures, molecular docking and fragment substitution results of CYP81BQ24, CYP81BQ23 and CYP81BQ19. **A** Alignment of the predicted 3D structures of CYP81BQ24, CYP81BQ23 and CYP81BQ19. Purple: CYP81BQ23, Orange: CYP81BQ24, Raspberry: CYP81BQ19. B Molecular docking results of CYP81BQ23. **C** Molecular docking results of CYP81BQ24. **D** Molecular docking results of CYP81BQ19. Amino residues from 91–140 were showed as cartoon, the ligand camptothecin was shown as a green stick structure (red represents O atom, blue represents N atom, green represents C atom) and heme as grey stick structure. The ten different amino acids are shown as stick structures, the amino acids that form hydrogen bonds with the substrate are shown as red stick structures, and the red dotted lines represent hydrogen bonds. **E** Fragment substitution results of CYP81BQ24 and CYP81BQ23

The sequence similarity of CYP81BQ24 with either CYP81BQ23 or CYP81BQ19 is over 90%. However, CYP81BQ24 and CYP81BQ23 catalyze the hydroxylation of CPT at different sites, while CYP81BQ24 and CYP81BQ19 exhibited different catalytic activity. To gain insight into regio-selectivity mechanism and activity enhancement of CPTHs, the protein structures were compared. Firstly, sequence alignment revealed that there were 51, 56 and 39 different amino acids between CYP81BQ23 and CYP81BQ24, CYP81BQ23 and CYP81BQ19, and CYP81BQ24 and CYP81BQ19, respectively (Figure S5). Then, the secondary structure element of substrate recognition sites (SRSs) was predicted. According to the standardized numbering scheme of CYP2A1, six putative SRSs were identified in the three proteins within the biggest difference concentrated in SRS1, SRS2, and SRS3. There is no difference in SRS4 and SRS5 between the three proteins. Finally, the three-dimensional structures of the proteins were predicted. Superposition of the proteins revealed highly similar structure (Fig. 4A). However, the position of substrate was not identical. Molecular docking showed that the binding sites of CYP81BQ24 and CYP81BQ19 to the substrates are close to each other, with CPT binds to CYP81BQ19 with a closer position to the heme ligand (Fig. 4C, D). Compared with CYP81BQ24 and CYP81BQ23, CPT binds to CYP81BQ23 with a position closer to the SRS1 region (Fig. 4B, C).

### Identification of a crucial region controlling CPT hydroxylation at C-10 or C-11 position

CYP81BQ24 and CYP81BQ23, which showed higher similarity were chose to elucidate the mechanism of regio-selectivity of CPT10H and CPT11H. According to the comparison of protein structures, the sequences were grouped into three fragments including fragment 1 (amino acid residue from 1–90), fragment 2 which contained SRS1, SRS2 and SRS3 (amino acid residue from 91–290) and fragment 3 which contained SRS4, SRS5 and SRS6 (amino acid residue from 291–512). Firstly, the three fragments of CYP81BQ23 and CYP81BQ24 were reciprocally exchanged. Replacement of fragment 1 of the two proteins (BQ23MF1 and BQ24MF1) does not change the function (Fig. 4E). Replacement of fragment 2 of CYP81BQ24 with that of CYP81BQ23 (BQ24MF2) resulted in the transformation from CPT 10-hydroxylation to 11-hydroxylation. However, replacement of fragment 2 of CYP81BQ23 with that of CYP81BQ24 (BQ23MF2) resulted in a complete loss of enzyme activity. Replacement of fragment 3 of CYP81BQ23 with that of CYP81BQ24 (BQ23MF3) did not change the function but resulted in an increase of catalytic efficiency. And replacing fragment 3 of CYP81BQ23 with that of CYP81BQ24 (BQ24MF3) resulted in a complete loss of enzyme activity. The results indicated that fragment 2 was important for the hydroxylation position of CPTHs.

A second round of reciprocal replacement was performed against fragment 2. Fragment 2 was divided into three fragments based on SRS1, SRS2, and SRS3, which were fragment 4 (amino acid residue from 91–140), fragment 5 (amino acid residue from 141–220) and fragment 6 (amino acid residue from 221–290). On the basis of BQ23MF2 and BQ24MF2, the replacement of fragment 5 and 6 were gradually reduced. Four mutants were constructed, BQ24MF4 and BQ23MF4 with replacement of fragment 4 and fragment 5, BQ24MF5 and BQ23MF5 with replacement of fragment 4. BQ24MF4 and BQ24MF5 were transformed to CPT 11-hydroxylation function, with the catalytic activity of BQ24MF4 stronger than BQ24MF5. However, no CPT hydroxylation function was characterized in BQ23MF4. A very small amount of CPT10H products was detected in BQ23MF5 (Fig. 4E). The result further narrowed the crucial region determining the CPT hydroxylation site to fragment 4 with a length of only 50 bp.

The third round of mutation were designed targeted CYP81BQ24 and CYP81BQ23, respectively. There are 11 different residues located in fragment 4 between CYP81BQ24 and CYP81BQ23. Based on SRS1, two mutants were designed for CYP81BQ24, BQ24MF6 with replacement of eight different residues located in SRS1 from CYP81BQ23 and BQ24MF7 with three different residues without SRS1. LC–MS results showed that both mutants retained CPT10H function (Fig. 4E). Combined the results of the two rounds of replacement and molecular docking, two mutants were designed for CYP81BQ23. BQ23MF6 was constructed with the replacement of amino acid residue of CYP81BQ24 from 91–431 and BQ23MF7 with the replacement of fragment 4 and fragment 7 (amino acid residue from 431–512) of CYP81BQ24. LC–MS analysis revealed that very small amounts of 10HCPT was detected under the catalysis reaction of BQ23MF6 while more 10HCPT was detected as products of BQ23MF7. Herein, the results validated that fragment 4 was a crucial region controlling CPT hydroxylation at C-10 or C-11 position. Alignment with the newly reported CPT10H (CYP81BQ18) reduced the number of divergent residues to ten. Thus, these results indicated that ten different amino acid residues located in fragment 4 of CYP81BQ23 and CYP81BQ24 dictate the regio-selectivity of CPT10H and CPT11H.

### Construction of a high-efficiency CPT 10-hydroxylase mutant

**Fig. 5:**
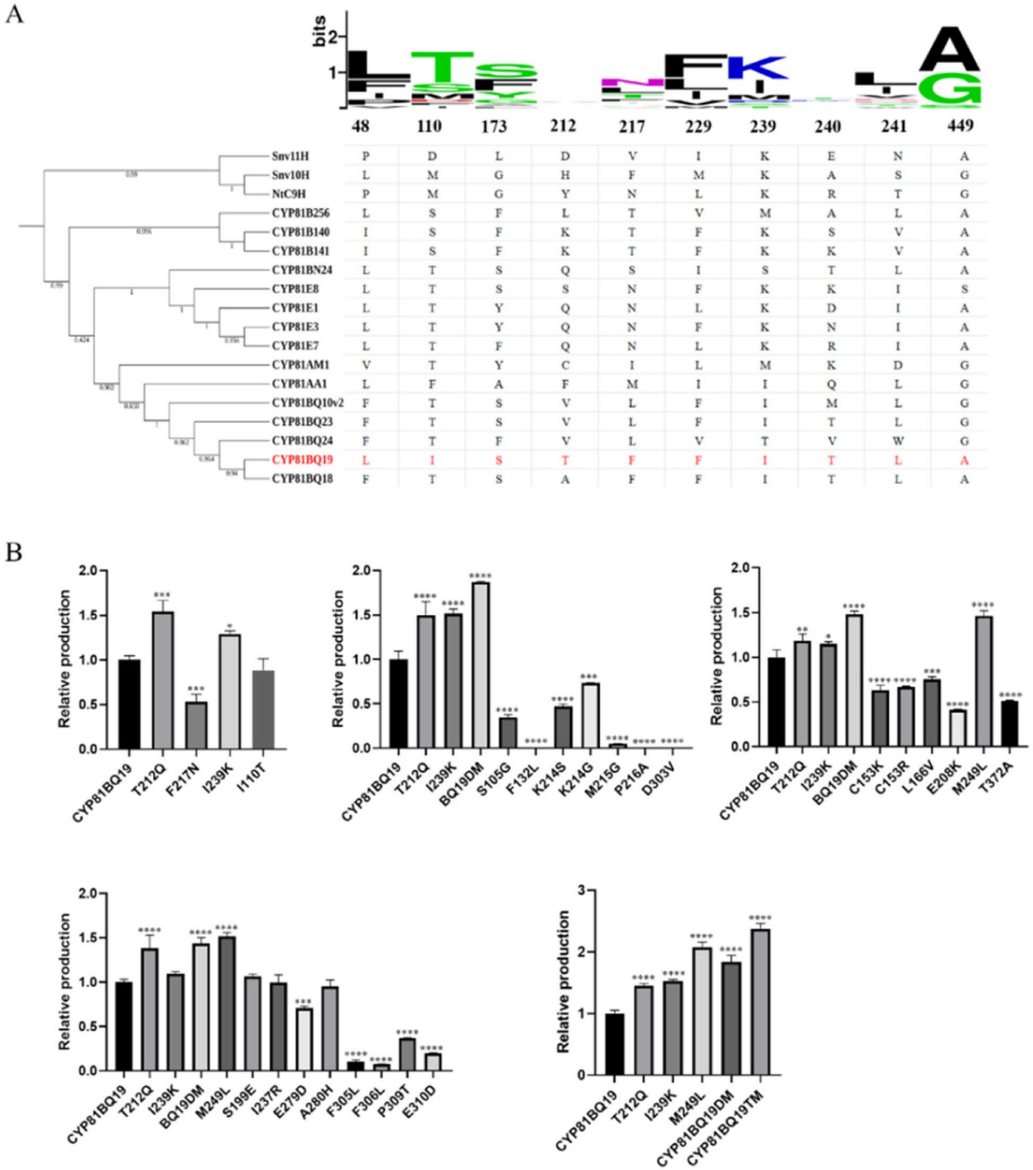
Construction of a high-efficiency mutant of camptothecin 10-hydroxylase. **A** Sequence conservation analysis of key different sites of CYP81BQ24 and CYP81BQ19. **B** Ultra-performance liquid chromatography (UPLC) detection of camptothecin 10-hydroxylase activity. * *P* < 0.05; ** P < 0.01, *** *P* < 0.001; **** *P* < 0.0001

Key amino acid residues for catalytic activity enhancement were screened by consensus analysis. A total of 18 sequences from CYP81, CYP82 and CYP71 families with or without hydroxylation functions were selected for alignments. Site-directed mutagenesis was designed using CYP81BQ19 as chassis. The first round of mutagenesis was performed by the comparison of CYP81BQ19 with CYP81BQ24. A total of 39 divergent amino acids were screened based on the sequence alignment of two proteins. Subsequently, seven residues were further targeted according to the positions of these amino acids on the three-dimensional structure (Fig. 5A). Residues around the entrance and binding pocket of substrate were selected. Consensus analysis of these seven residues within 18 sequences showed that four amino acids of CYP81BQ19 were not the conserved ones including I110, T212, F217, I239. Therefore, four mutants were constructed in the first round of mutagenesis correspondingly, which included CYP81BQ19^I110T^, CYP81BQ19^T212Q^, CYP81BQ19^F217N^ and CYP81BQ19^I239K^. UPLC analysis revealed CYP81BQ19^T212Q^ and CYP81BQ19^I239K^ resulted in significant increase of the production of 10HCPT, with that of CYP81BQ19^T212Q^ yield by 54.66% and CYP81BQ19^I239K^ yield by 28.95% (Fig. 5B). CYP81BQ19^I110T^ and CYP81BQ19^F217N^ resulted in dropped products. The second round of candidate sites included residues distributed within 4 Å of the substrate and residues located between 140–290 of the protein. Sites for mutation were downsized to 21 based on consensus analysis, which included G105, L132, K/R153, V166, E199, K208, S/G214, G215, A216, R237, L249, D279, H280, V303, L305, L306, T309, D310 and A372 (Figure S6). The construction and functional characterization of all the site-directed mutants were arbitrarily grouped into four batches. Only one mutant CYP81BQ19^M249L^ showed 51.57% improvement in production (Fig. 5B). All the other 20 mutants resulted in a reduction of products. Then, iterative mutations were performed by introducing K239 and L249 successively into the CYP81BQ19^T212Q^ chassis. The production of CPT10H catalyzed by CYP81BQ19DM and CYP81BQ19TM increased 84% and 138%, respectively.

## Discussion

### DIMORPH is a pioneering database and analytical platform centered on CPT

In this study, a multi-omics database centered on CPT was established. In addition to collecting published data, we also generated and obtained new data. Although published omics data of CPT-producing plants is abundant, newly generated datasets established some gaps in the current literature. Compared with researches, this study provided the first publicly accessible sequence dataset for the genome of *N. nimmoniana*. And the transcriptome data of *C. acuminata* is the only published available data that used the same batch of samples from different tissues for metabolome detection (Sadre et al. 2016; Pu et al. 2022). Moreover, the dataset was sampled from plants grown in the same place, in the same growth stage, and with each tissue sample having three replicates. While the samples for data referenced in current literature (Góngora-Castillo et al. 2012) were from plants in three regions, at different growth stage, and there was only one sample for each tissue except for the mature leaf. Therefore, newly generated dataset is a good supplement.

To the best of our knowledge, DIMORPH is the first database centered on a single compound. Existing databases are instead focused on one specific species (Xiao et al. 2021), species in family level (Wang et al. 2020), or collecting as many genomes as possible (He et al. 2022; Liao et al. 2022; Xia et al. 2019). Here, DIMORPH targeted the important anti-tumor component, CPT. All of the data contained in the database are related to it. Multiple omics data from the three representative CPT-producing plants were collected rather than having a database containing as many species as possible. Key metabolites involved in the biosynthetic pathway of CPT were integrated rather than untargeted metabolome detection data. Additionally, a specific module for the biosynthetic pathway of CPT was designed, in which all relevant omics data was linked. Considering the high value of CPT in clinical settings and high market demand, construction of such a database is significant as it filtered and selected data, providing easy access to CPT-related candidates. Secondly, DIMORPH aids the users interested in CPT without sufficient bioinformatic background as all data, alongside analytical results have been prepared in a user-friendly interface. For example, if researchers are interested in a gene, they can clearly browse all the basic information in the gene module by inputting the gene ID or sequence in DIMORPH instead of downloading the sequence and annotation files from public databases and input instructions to extract that gene with location, gene length, and sequence. Thirdly, DIMORPH contains analytical data, including the functional annotation, co-expression network construction and genome comparison. Users can simply click on the corresponding module to get the results directly, without having to run through many procedures. For example, regulatory mechanism of the CPT biosynthesis is a hotspot (Hu et al. 2020; Hao et al. 2023). DIMORPH provided whole-genome analysis results of transcription factors, which accelerated research progress by avoiding tedious pre-analysis work. DIMORPH has also provided a simple-navigating interface of functional tools for users to do some analysis and comparison of their own data. In particular, DIMORPH can illustrate the worldwide attention paid to CPT with a tool for real-time monitoring visits to the website. Based on statistics, users will get an idea of how many people across many regions around the world are interested in CPT. Undoubtedly, it will accelerate the process of CPT-related research.

However, with the development of next generation sequencing, more and more multi-omics data of CPT-containing plants was decoded extensively, such as the proteome and single-cell transcriptome of *C. acuminata* (Wang et al. 2024b; Zhang et al. 2023a, b), as well as the transcriptome of *Nothapodytes tomentosa* (Chen et al. 2024). Due to reasons such as non-publication or download restrictions, these data were not included in the database. To address this issue, a data update alert system will be established in the backend of the database, enabling real-time monitoring of newly available resources for timely updates. Furthermore, the database will develop a user submission portal to support the upload of relevant data by users in the future.

### DIMORPH facilitated the characterization of a novel CPT 10-hydroxylase in *C. acuminata*

A new CPT10H, CYP81BQ24, was discovered and identified from *C. acuminata* in this study. Notably, DIMORPH played a key role in the discovery of CYP81BQ24. The biosynthetic pathway of the CPT module pointed to the incorrect annotation of two genes, *CacGene08149* and *CacGene27004*. Based on newly generated transcriptome data, both genes were reannotated and subsequent cloning experiments confirmed the accuracy of the revised gene structures. This may not be an isolated case in the genome of *C. acuminata*. Previous research showed that two functional genes, *CYP81BQ18* (*CacGene08146*) and *CYP81B257* (*CacGene01301*) shared only 73.54% and 47.79% sequence identity, respectively (Pu et al. 2024), with their corresponding genes in the genome of *C. acuminata*. The low sequence identity was attributed to variations in sequence length as segments of both genes exhibited 100% sequence identity with their counterparts. It is speculated that the annotation of these corresponding genes may also be incorrect, similar to *CacGene08149* and *CacGene27004*. In fact, varying degrees of gene absence and incompleteness were observed across the genomes, even for identified genes. Research has shown that high-quality assembly, such as T2T assembly, significantly improves completeness and accuracy (Cheng et al. 2025). The precise localization of functional genes in the genome provided a clear roadmap for natural product biosynthesis. Based on the reannotated results, CYP81BQ24 was screened and functionally characterized. The sequence and chromosomal location of *CYP81BQ24* differ from those of *CYP81BQ23* and *CYP81BQ19* (Nguyen et al. 2021), which confirmed that *CYP81BQ24* is a novel CPTH. Additionally, *CYP81BQ24* and *CYP81BQ19* were located in close proximity on the same contig of the genome assembly, suggesting they may be tandemly duplicated genes involved in the biosynthesis of 10HCPT. To date, four CPT10Hs have been reported (CYP81BQ24, CYP81BQ19, CYP81BQ18, and CYP81B256), however, it remains unclear which of these is the key one responsible for 10-hydroxycamptothecin formation (Nguyen et al. 2021; Pu et al. 2024). Previous studies have shown that silencing CYP81B256 and CYP81BQ18 in *C. acuminata* plantlets using VIGS resulted in 66% and 61% reductions in transcript levels, respectively, accompanied by decreases in 10-hydroxycamptothecin content by 36.7% and 32.3% (Pu et al. 2024). These findings indicate that multiple CPT10Hs likely contribute to 10-hydroxycamptothecin biosynthesis in *C. acuminata*. Moreover, a similar situation is observed for other key genes involved in the biosynthetic pathway of CPT, such as CaSLSs and Ca7DLHs, for which the dominant functional members have not yet been clearly identified (Liu et al. 2024a, b; Yang et al. 2019). Therefore, subsequent studies should be designed to elucidate the biosynthetic mechanism of CPT in *C. acuminata*. DIMORPH provided a valuable platform for the selection of functional candidate genes, laying a foundation for elucidating the biosynthetic mechanisms of CPT in plants.

### Ten key residues determined the regio-selectivity of CPTHs at C-10 and C-11 position

The hydroxylation products at different positions of CPT exhibit varying inhibitory effects on different cancer cells (Wall et al. 1986). Consequently, it is crucial to analyze the key residues of CPTHs. Key amino acids led to the exquisite selectivity of NtCPT9H in the C-9 hydroxylation of CPT has been elucidated (Chen et al. 2024). However, the crucial amino acids responsible for the C-10 and C-11 hydroxylation of CPT remain unclear. The identification of CYP81BQ24 has facilitated the exploration of the selectivity determinants of CPT10H and CPT11H, as it shows the highest sequence similarity (90.04%) to CYP81BQ23 among all the CPT10Hs reported. Therefore, CYP81BQ24 represents an ideal model for investigating the key amino acid residues. Through comparative analysis of CYP81BQ23 and CYP81BQ24 followed by stepwise fragment replacement, ten amino acids located in fragment 4 were identified as the key residues responsible for the regio-selectivity of CPT10H and CPT11H. Since the function of BQ24MF6 and BQ24MF7 did not change, it suggested that residues in the preceding and following segments of this region are critical. It is possible that the regio-selectivity is determined by the combined action of all ten residues. It is mentioned that most critical residues are located in the key areas located in SRS1 region. Several factors may account for this distribution. Firstly, sequence alignment results showed that CYP81BQ23 and CYP81BQ24 exhibited significant differences between SRS1, SRS2, and SRS3 regions, with a total of 14 different amino acid residues. Notably, SRS1 contains most different amino acid residues (up to eight sites), indicating that this region may have the most significant influence on the CPT 10- and 11-hydroxylation function. Furthermore, protein structure prediction and molecular docking analyses indicated that the substrate binds in close proximity to SRS1 in CYP81BQ23, further implying that the functional divergence is likely governed by key residues located in the SRS1 fragment. We believe that the conclusion regarding key regulatory regions of CPT10H and CPT11H remains applicable to other CPT10Hs including CYP81BQ19 and CYP81BQ18. This is supported by analysis from three aspects. Firstly, phylogenetic analysis revealed that CYP81BQ19 and CYP81BQ18 were classified into the same clade with CYP81BQ24 and CYP81BQ23 (Figure S2), sharing over 88% sequence identity. Secondly, like CYP81BQ24, the most divergent region between CYP81BQ19/CYP81BQ18 and CYP81BQ23 is located within amino acid residues 91–140, the key regulatory region. Thirdly, the ten key amino acid residues within this region are conserved between CYP81BQ19, CYP81BQ18, and CYP81BQ24. Therefore, these findings support that the conclusion regarding the key region determining catalytic function is applicable to CYP81BQ19 and CYP81BQ18. Whether the same conclusion applies to CYP81B256, however, remains an open and intriguing question. Although CYP81B256 is phylogenetically close to CYP81BQ24 (Figure S2), it shares only 48% sequence identity. It is generally accepted that proteins with low sequence similarity but similar functions often employ distinct catalytic mechanisms. Nevertheless, exceptions do exist. For example, although AlkB shares limited overall sequence and structural similarity with other lipid-metabolizing proteins in FADS-like superfamily, signature motifs and residues suggest a certain degree of shared catalytic mechanism among these nitrogen-rich diiron centers (Guo et al. 2023). Similarly, Zhang et al. identified key residues in substrate recognition and selective hydroxylation in CYP109B4, which was also applicable to other family members including CYP109B3, despite only 55% sequence identity (Zhang et al. 2023a, b). These examples imply that a similar mechanism of selectivity control mediated by key regions may exist across divergent members of the same cytochrome P450 family. Therefore, it represented a promising avenue for future research.

It is also noted that certain mutants (i.e. BQ23MF2), despite carrying the fragment encompassing all ten key residues, they failed to exhibit the expected function. This may be related to catalytic efficiency. On the one hand, the catalytic activity of CYP81BQ24 is lower than that of CYP81BQ23. Therefore, it was difficult to obtain the function of CYP81BQ24 by replacing fragments from CYP81BQ24. And the other, the results from BQ24MF2, BQ24MF4, and BQ24MF5 indicated that replacing fragments 5 and 6 from CYP81BQ23 significantly improved catalytic activity. As a result, although BQ23MF2 contained the critical ten residues for obtaining CPT10H function, no product was detected because it also contained fragments 5 and 6, which reduced catalytic activity. A similar phenomenon has been reported in other studies. Islam et al. reported that a sextuple mutant of 1,3,5-trihydroxyxanthone synthase released 91.1% of the total product as 1,3,7-trihydroxyxanthone from 1,3,5-trihydroxyxanthone. However, reciprocal substitution from 1,3,7-trihydroxyxanthone synthase to 1,3,5-trihydroxyxanthone synthase had only minor effects, and most mutants lost enzyme activity (El-Awaad et al. 2016). Liu et al. reported that diterpene synthases AfAS and fusicocca-2,10(14)-diene synthase share the same active site residues at all positions except position 65. However, the substitution of residue 65 could only generate the function of PaFS. Further saturation mutagenesis resulted in the function of AfAS (Liu et al. 2024a, b). Therefore, the regio-selectivity mechanism of these proteins is complex. As for CPTHs, future studies may further explore key residues through site-directed mutagenesis or crystallographic analysis of CYP81BQ24 and CYP81BQ23 to elucidate the molecular determinants underlying their functional specificity.

### The construction of a high-efficiency CPT10H mutant laid the foundation for the biosynthesis of CPT-derived drugs

10HCPT is the important precursor of CPT-based anticancer drugs. It is well known that irinotecan and topotecan were semi-synthesized from 10HCPT. However, the accumulation of 10HCPT in plants was extremely low, highlighting the urgent need for an efficient CPT10H enzyme for the biosynthesis of CPT-derived drugs. Considering the catalytic activity of CYP81BQ19 is higher than CYP81BQ24 and other CPT10Hs reported, we selected CYP81BQ19 as the chassis in order to obtain a high-efficiency mutant of CPT10H. As for CYP81BQ24, it played an important role in the construction of a high-efficiency CPT 10-hydroxylase mutant. Two rounds of mutagenesis were carried out to enhance catalytic efficiency. The first round of mutagenesis was performed by the comparison of CYP81BQ19 with CYP81BQ24. Since both proteins perform the same function, their different amino acids are bound to affect the catalytic efficiency. Molecular docking and consensus analysis further narrow down key residues to four. In result, two (CYP81BQ19^T212Q^ and CYP81BQ19^I239K^) out of the four mutants could significantly improve the catalytic activity. The second-round of mutagenesis expanded the range based on the results of molecular docking and previous fragment replacement. All the residues distributed within 4 Å of the substrate were included. Besides, fragment replacement results indicated that the fragment 4 and 5, namely residues located in 141–290 of CYP81BQ24 caused decreased activity. Thus, amino acids located in this region with the highest probability was chosen for mutation reference. In contrast, the results were less promising. During the second round of mutagenesis, where the screening scope was expanded without relying on the comparison of CYP81BQ19 with CYP81BQ24, only one (CYP81BQ19^M249L^) out of 21 mutants exhibited a significant improvement in activity. These results indicated that comparative analysis with CYP81BQ24 enabled the efficient identification of key residues that enhance catalytic activity, thereby markedly improving the efficiency of the construction of high-efficiency mutant.

Mutation of all three targeted residues yielded a highly efficient mutant, CYP81BQ19TM, with a 138% increase in catalytic efficiency relative to the wild-type enzyme. Although the improvement in catalytic efficiency achieved in this study is modest relative to other enzyme engineering efforts based on similar strategies, the inherent complexity of plant CYP450 modification underscores the significance of this achievement (Li et al. 2024; Wang et al. 2024a). Besides, since most plant CYP450s are membrane-bound proteins, they are difficult to express in prokaryotic systems and challenging to purify in eukaryotic systems, so the kinetic parameters such as Kcat and Km are difficult to calculate (Sun et al. 2020). As a result, the catalytic activities of plant CYP450s are commonly assessed by comparing the quantity of target products in engineered yeast. For instance, Sun et al. evaluated the oxidation activity of CYP72A63 mutants by measuring product quantity in yeast fermentation (Sun et al. 2020); Liu et al. compared the activities of two homologous hydroxylases based on product quantity in yeast (Liu et al. 2017); and Mao et al. screened for high-efficiency mutants of CYP76AH1 and CYP76AH3 by comparing differences in product yields in yeast (Mao et al. 2020). Given the difficulty in purifying plant CYP450 proteins, our study also relied on the quantification of 10-hydroxycamptothecin produced in yeast to compare the catalytic activity.

The significantly altered physicochemical properties of the mutant residues might be the main factor contributing to the enhancement of activity. Molecular docking results suggested two possible mechanisms. First, T212 and I239, located in the key region of the substrate binding pocket, are thought to enlarge the entrance to the substrate channel (Figure S7). It is a common method for engineering modification of CYP450 family enzymes by regulating the substrate channel to improve catalytic efficiency (Meng et al. 2022). And the other, the substrate binding to CYP81BQ19TM can form hydrogen bonds with heme, potentially enhancing the hydroxylation efficiency by optimizing the electron transfer pathway. However, the mechanism underlying the significant improvement in catalytic efficiency due to the M249 mutation remains unclear, as it is located far from the active center. We speculate that M249 may play a role as a key node in the protein's regulatory network as the catalytic mechanism of proteins was complex. For example, research demonstrate a pivotal switch in β-domain determines the substrate selectivity of terpene synthases involved in *Gardenia jasminoides* floral scent synthesis, which was previously deemed “non-functional” region (Chen et al. 2025). To further elucidate the mechanism, it will be essential to obtain the protein crystal structure or conduct additional research using molecular dynamics simulation software.

## Conclusion

In summary, a comprehensive database integrating multiple omics data of CPT-producing plants was constructed in this study, providing a valuable platform for CPT-related researches. Based on this platform, a novel CPT10H, CYP81BQ24, was identified and functionally characterized in *C. acuminata*. Comparative analysis between CYP81BQ24 and CYP81BQ23 revealed ten critical residues responsible for catalyzing 10- and 11-hydroxylation of CPT. Moreover, a high-efficiency CPT10H mutant was obtained through site-directed mutagenesis. This work provided important insights into the CPT biosynthetic pathway and offered a solid foundation for future efforts in the synthetic biology of CPT-derived therapeutics.

## Materials and methods

### Data collection

Multi-omics data of *C. acuminata*, *O. pumila* and *N. nimmoniana* was generated in this study and collected from public databases and published studies (Table 1). The following is a detailed description of the data collection methods.

#### Newly generated data

Plant materials. *C. acuminata* plants were collected from two regions, the Botanical Garden of Xishuangbanna South Medicine, Yunnan, China and Dangyang, Hubei province, China. The plants were identified by Prof. Li Haitao (Yunnan Branch, Institute of Medicinal Plant, Chinese Academy of Medicinal Sciences). Six independent organs (Up stem, Mid stem, Low stem, Root, Young leaf and Mature leaf) from *C. acuminata* were collected in three replicates. *O. pumila* plants were collected from wild plants growing in Guangzhou, Guangdong Province, China, which were authenticated by Prof. Lin Yulin (Institute of Medicinal Plant Development, Chinese Academy of Medical Sciences). Four independent tissues (Sem, Root, Leaf and Fruit) from *O. pumila* were collected in three replicates. The young leaf of *N. nimmoniana* were collected from Jinghong city, Xishuangbanna Dai Autonomous Prefecture, Yunan Province, China, which were identified by Prof. Li Haitao (Yunnan Branch, Institute of Medicinal Plant, Chinese Academy of Medicinal Sciences).

Sample preparation and sequencing*.* High-quality DNA was extracted from young leaves of *N. nimmoniana* and sequenced by Illumina HiSeq 4000 platform. Total RNA was extracted from different tissues of *C. acuminata* and *O. pumila* using an RNAprep Pure Plant kit (Tiangen, China). The isolation, enrichment, and fragmentation of mRNA as well as the synthesis of the first and second strand were performed using a TruSeq RNA Library Prep Kit v.2 (Illumina, USA) and the purification was performed using a QiaQuick PCR kit (Qiagen, Germany). Libraries were then sequenced using an Illumina Novaseq platform. The raw sequencing data were trimmed with quality control metrics including removing adapter, poly-N sequences and low-quality reads (base quality score ≥ 20) using TRIMMOMATIC (v.0.39), and the clean data were de novo assembled using TRINITY (v.2.8.3). Fragments per kilobase of exon model per million reads mapped (FPKM) values for RNA-Seq reads were calculated using both HISAT2 (v.2.0.5) and CUFFLINKS (v.2.2.1).

Detection of metabolites participating in CPT biosynthesis. For each sample, 100 mg of powdered tissue was extracted using 1 mL acetonitrile/water (7/3, v/v). Samples were vortexed for 5 min, and ultrasonicated for 30 min. Then the samples were centrifuged at 12,000 × *g* at 4 ℃ for 20 min. And 100 μL of the supernatants were vortexed with 10 μL Carbamazepine (25 μg/mL) as internal standard for quality control. Quantitative analyses of metabolites involved in the biosynthesis of CPT were carried out by liquid chromatography-mass spectrometry (Agilent Technologies 1290 Infinity II LC System and 6545 Q-TOF, with the electron spray ionization ion source operated in positive ion mode (50–800 m*/z*). An Agilent Zorbax SB C18 (100 × 2.1 mm, 3.5 μm) was used at 35 °C, with water containing 0.1% formic acid (A) and acetonitrile (B) as mobile phases. Separation was performed at a flow rate of 0.3 mL min^−1^ with the following gradient: 0–2 min, 5% B; 2–20 min, linear increase from 5 to 20% B; 20–43 min, linear increase from 20 to 55% B, 43–50 min, linear increase from 55 to 90% B. The auxiliary gas, sweep gas and sheath gas was N_2_. Data were analyzed with Xcalibur 2.0 SR2 (Thermo Fisher Scientific).

#### Data downloaded from public databases and published studies

Assembled genome of *C. acuminata* was downloaded from Figshare database (Kang et al. 2021) and Dryad Digital Repository (Zhao et al. 2017). Assembled genome of *O. pumila* was downloaded from National Center for Biotechnology Information and a dedicated server (http://pumila.kazusa.or.jp/) (Rai et al. 2021). Transcriptome of *C. acuminata* (Zhao et al. 2017) and *N. nimmoniana* (Rather et al. 2018) was downloaded from references. Metabolomic data of the three species was downloaded from references (Namdeo et al., 2012; Rai et al. 2021; Sadre et al. 2016; Wu et al. 2008; Yamazaki et al. 2013).

### Omics data analysis and integration

All data were categorized into three fields: genome, transcriptome and metabolome. Data were inspected and confirmed to the same format if available.

#### Genome

A total of three assembled genomes were integrated in DIMORPH (Table 1). Functional annotations of all genomes were executed through a unified procedure by Non-Redundant Protein Sequence (NR), Gene Ontology (GO), Kyoto Encyclopedia of Genes and Genomes (KEGG), Swiss-Prot and Pfam databases. Genes belonging to the cytochrome P450 (CYP450) and transcriptional factor (TF) gene families were classified. Among them, genes classified to the CYP450 family were annotated with the conserved PFAM domain of PF00067 and TFs were predicted by Plant Transcription Factor Database (Guo et al. 2008). Comparative genome analysis between any two genomes in DIMORPH was performed by MCScanX.

#### Transcriptome

Three assembled transcriptomes were integrated in DIMORPH (Table 1). In total, transcriptome expression profiling data of 48 samples were collected including 37 samples from *C. acuminata*, nine samples from *O. pumila*, and two samples from *N. nimmoniana* (Table S1). The expression data of each genome of *C. acuminata* consists of two sets, one of which is derived from 19 samples and is recalculated using the RNA-seq data in a previous study based on these two genomes (Góngora-Castillo et al. 2012; Kang et al. 2021; Zhao et al. 2017). The other set of data was obtained using the samples collected in this study. A co-expression network of all 37 samples from *C. acuminata* was analyzed using Weighed Correlation Network Analysis (WGCNA).

#### Metabolome

Detailed data including chemical formulas, MS/MS fragment profiles and accumulation levels in different tissues of 15 metabolites involved in the CPT biosynthetic pathway were collected. The chemical structural formulas were drawn using KingDraw software. The MS/MS fragment profiles were collected from prior studies (Table S2) (Jin et al. 2019; Sadre et al. 2016; Yang et al. 2021). A total of 66 samples were included in DIMORPH with the data of accumulation levels in different tissues, including 36 samples from *C. acuminata*, which were generated in the study, and 30 samples from *O. pumila*, which were obtained from previous studies (Rai et al. 2021).

### Database construction

The database was deployed in the Ubuntu 16.04 operating system and developed by AKKA 2.6.5(web server), MySQL 5.7(database server), Scala 2.13.2 and SBT 1.3.9. All data in the database were managed and stored by MySQL Database Management System. The query function was enforced based on the Slick 3.3.2 middleware tier. JBrowser 1.16.6 was embedded to visualize each genome. The website interface components were designed and implemented by Bootstrap 4.6.0, Vue 3.0.4, and Play Framework 2.8.7. The website has been tested in several popular web browsers, including Firefox, Google Chrome, and Internet Explorer. Source code is available in gitee (https://gitee.com/vg_soft_team_rep/camptothecin_derived_db).

### Cloning and sequencing of reannotated genes

Total RNA of *C. acuminata* from six tissues were reverse transcribed using the PrimeScript™ II 1 st Strand cDNA Synthesis Kit (Takara, China) to synthesize one strand of cDNA. Primers used for clone were listed in Table S3. Coding sequences of *CYP81BQ23*, *CYP81BQ24*, *CYP81BQ10v2*, *CYP81BQ19* and *CYP81BQ25P* were cloned from cDNA and the genomic sequence (2,799 bp) including the coding region of *CYP81BQ24* was cloned from DNA. The amplified products were ligated into the pMD18-T vectors (Takara, China) and transformed into *Escherichia coli* DH5α cells (Tiangen, China). Then, the plasmids were extracted for Sanger sequencing. Cloned coding sequence was translated to amino acid sequences and then submitted to Prof. Nelson for standard nomenclature (Nelson 2006).

### Heterologous expression of CYP81BQ24

The coding sequences of *CYP81BQ24*, *CYP81BQ23* and *CYP81BQ19* were cloned into pYES2-CT vector and transformed into *Saccharomyces cerevisiae* strain WAT11 using LiAc/PEG method (Gao et al. 2022). *CYP81BQ23* and *CYP81BQ19* were transformed as positive control and the empty vector of pYES2-CT was transformed as negative control. The colonies were picked and confirmed by colony polymerase chain reaction using the GAL1F/R common primers to verify the plasmids were successfully transferred into the yeast cells.

### In vivo enzyme assay of CYP81BQ24

In vivo functional characterization was performed under standard conditions. The engineered strains were first grown in 4 mL SD-Ura medium containing 2% glucose at 30 ℃ for 24 h. The culture was then diluted to an OD600 of 0.05 in SD-Ura medium and cultured for 16 h. The yeast cells were harvested by centrifugation at 1000 × *g* for 5 min, and resuspended the culture with YPA medium (2% peptone, 1% yeast extract, 10% glycerol, 2% galactose). Subsequently, 1 mL culture were transferred to a cell culture tube, and 10 μM CPT were fed. Each reaction was performed in triplicate. After 48 h incubation, the reaction mixture was terminated with 1 mL ethyl acetate twice. The upper layer was transferred and dried using vacuum concentration at 30 ℃, 1100 rpm. The residue was diluted with 120 μL methanol and filtered through a filter membrane (0.22 μm).

### In vitro enzyme assay of CYP81BQ24

The microsomes were obtained as previously described (Nett et al. 2020). In brief, positive colonies were inoculated in 4 mL SD-Ura medium at 30 ℃ for 2 days. Then, 2 mL of the initial culture was used to inoculate 200 mL of YPGE medium. Cultures were grown overnight at 30 ℃ until reaching platform, at which point a sterile aqueous galactose solution was added to a final concentration of 5% (v/v). After 12 h induction, cultures were centrifuged. Cell pellets were crushed with glass beads (0.5 mm diameter) mechanically by vigorous up- and—down shaking for 5 min (30 s of shaking followed by 30 s on ice). The supernatant was carefully transferred and microsomes were precipitated by the addition of NaCl and polyethylene glycol (PEG)−4000. The microsomal protein fractions were resuspended in 1 mL of ice-cold TEG storage buffer after centrifugation at 10,000 × *g* for 10 min at 4 °C. Microsomal protein content was quantified using the BradFold Protein Assay Kit (Beyotime, China).

Microsomal enzyme assays were performed at 30 ℃ for 2 h in 100 μL of reaction buffer (100 mM Tris, pH 7.5) containing 50 μM substrate, 10 μM FAD, 10 μM FMN, 600 μM NADPH, 5 mM glucose-6-phosphate, 1 U glucose-6-phosphate dehydrogenase, and 0.1 mg microsomal protein. The reaction mixture was terminated with 200 μL of ethyl acetate and centrifuged. The upper layer was transferred and vacuum concentrated. The residue was diluted with 120 μL methanol and filtrated through a filter membrane (0.22 μm).

### LC–MS analysis

The reaction was analyzed by a liquid chromatography-mass spectrometry (Agilent Technologies Palo Alto, CA, USA 1290 Infinity II and 6545 Q-TOF, with the Dual Agilent Jet Stream Electrospray Ionization ion source) operated in positive ion mode (mass range: 50–1000 m*/z*). An Ultimate XB C18 column (3 µm, 2.1 × 100 mm) was used at 40 °C, with water containing 0.1% formic acid (A) and acetonitrile (B) as the mobile phases. Separation was performed at a flow rate of 0.25 mL/min with the following gradient: 0–2 min, 5% B; 2–4 min, 5–12% B; 4–15 min, 12–25% B; 15–17 min, 25–95% B; 17–19 min, 95%B; 19–19.5 min, 5%B. Data was analyzed with Agilent MassHunter (10.0).

### Optimization of in vivo reaction conditions

The optimum reaction conditions for CYP81BQ24 were determined through a series of experiments including reaction time (24, 48, 72, 96, 120 h), temperature (25, 30, 35℃), pH (4.0, 5.0, 6.0, 7.0, 8.0, 9.0), volume (500 μL, 1, 5, 10, 20 mL), carbon sources (10% glycerol, 2% glucose + 10% glycerin, 2% galactose + 10% glycerin, 2% sucrose + 10% glycerin, 2% trehalose + 10% glycerin) and substrate concentration (10, 20, 50, 100, 200 and 500 μM). Each experiment was performed with single factor changed under standard conditions described above. Each experiment was repeat three times.

### Comparison of the catalytic activity of CPT10Hs

The sequence of CYP81BQ18 and CYP81B256 were downloaded from reference (Pu et al. 2024) and synthesized to pYES2-CT vector by Genscript Biotech Co. Ltd (China). Heterologous expression and in vivo enzyme assay were performed as described above.

### Structure prediction and molecular docking

The secondary structures of CPTHs were predicted by aligning with previously established SRS region in CYP2A1 (GenBank: P11711). The three-dimensional structure of CPTHs was predicted using Alphafold 2. Heme and CPT was downloaded in PDB format from the PubChem Data Bank. The docking of proteins and substrates was determined using AutoDock Vina program (V1.5.7). The three-dimensional structure and molecular docking results were visualized using Pymol (V2.4.1).

### Fragmental and site-directed mutagenesis

Partially overlapping complementary primers were designed. Target sequences were amplified and homologous recombined using Mut Express II Fast Mutagenesis Kit V2 (Vazyme Biotech, China). Recombinant products were transformed to *E. coli* DH5a competent cells (Tiangen Biotech, China). Plasmids were isolated and sequenced to confirm the substitution of fragments or the designed mutations.

### Functional identification of mutants

Heterologous expression and functional characterization were performed as described above. The reaction conditions of mutants were optimized. These in vivo assays were performed at 30 ℃ in 1 mL of the strain (pH 8.0) for 72 h, feeding with 20 μM substrate and using 2% galactose + 10% glycerin as carbon source. The products of fragmental mutagenesis were analyzed using the liquid chromatography-mass spectrometry. The products of site-directed mutagenesis were performed on Waters ACQUITY UPLC system with an Acquity UPLC BEH C18 column (1.7 µm, 2.1 × 100 mm). The mobile phase was composed of A (water and 0.1% formic acid) and B (acetonitrile) using a gradient elution of 5% B at 0–2 min, 5–21% B at 2–4 min, 21–25% B at 4–14 min, 25–45% B at 14–16 min, 45–95% B at 16–18 min, 95–5% B at 18–20 min and 5% B at 20–21 min. Further, the flow rate of the mobile phase was set at 0.25 mL/min. The column temperature was conditioned at 30 °C and absorption was detected at 265 nm.

## Supplementary Information

Supplementary file 1. Figure S1 Alignment of the newly generated raw transcriptome data of *Camptotheca acuminata* with*CacGene27004* and *CacGene08149*. Figure S2 Phylogenetic relationship of CYP450s in the genome of *Camptotheca acuminata*. Figure S3 The MS spectra, ultraviolet absorption peak of the products of camptothecin hydroxylases. (A) Characteristic cleavages. (B) Ultraviolet absorption peak. (C) Extracted ion chromatograms from LC-MS analysis of *in vitro* reactions catalyzed by CYP81BQ24. Figure S4 The comparison of catalytic activity of CPT 10-hydroxylases *in vivo.* *P<0.05; ** *P*<0.01, *** *P*<0.001; **** *P*<0.0001. Figure S5 Sequence alignment of CYP81BQ24, CYP81BQ19 and CYP81BQ23 and identification of substrate recognition sites. Figure S6 Sequence conservation analysis of CYP81BQ19 key differential sites in a wide range. Figure S7 Molecular docking and substrate entrance of CYP81BQ19 and CYP81BQ19TM. (A) CYP81BQ19. (B) CYP81BQ19TM. Table S1 RNA samples used in this study. Table S2 MS/MS fragment profiles of key metabolites involved in the camptothecin biosynthetic pathway. Table S3 Primers for the clone of reannotated genes. Table S4 Summary of functional annotation results. Table S5 Distribution of transcriptional factors in the genomes of *Camptotheca acuminata* and *Ophiorrhiza pumila*. Table S6 Summary of comparative genome analysis results in DIMORPH.

## Data Availability

The authors confirm that all data in the study are included in this article (and its supplementary information file).

## Abbreviations

CPT: Camptothecin
DIMORPH: Database integrated multiple omics resources for CPT-producing herbs
CPTH: Camptothecin hydroxylase
CPT10H: Camptothecin 10hydroxylase
CPT11H: Camptothecin 11-hydroxylase
CYP450: Cytochrome P450
CDS: Coding sequence
GO: Gene ontology
KEGG: Kyoto encyclopedia of genes and genomes
