# Supplementary material for: DIMORPH: an integrated multi-omics resource for camptothecin-producing plants
Source: Mol Hortic. 2026 Jun 3;6:40. doi: 10.1186/s43897-025-00225-4 (PMC13231748; doi:10.1186/s43897-025-00225-4)
Supplement: Supplementary file 1 — Figure S1 Alignment of the newly generated raw transcriptome data of Camptotheca acuminata withCacGene27004 and CacGene08149. Figure S2 Phylogenetic relationship of CYP450s in the genome of Camptotheca acuminata. Figure S3 The MS spectra, ultraviolet absorption peak of the products of camptothecin hydroxylases. (A) Characteristic cleavages. (B) Ultraviolet absorption peak. (C) Extracted ion chromatograms from LC-MS analysis of in vitro reactions catalyzed by CYP81BQ24. Figure S4 The comparison of catalytic activity of CPT 10-hydroxylases in vivo. *P<0.05; ** P<0.01, *** P<0.001; **** P<0.0001. Figure S5 Sequence alignment of CYP81BQ24, CYP81BQ19 and CYP81BQ23 and identification of substrate recognition sites. Figure S6 Sequence conservation analysis of CYP81BQ19 key differential sites in a wide range. Figure S7 Molecular docking and substrate entrance of CYP81BQ19 and CYP81BQ19TM. (A) CYP81BQ19. (B) CYP81BQ19TM. Table S1 RNA samples used in this study. Table S2 MS/MS fragment profiles of key metabolites involved in the camptothecin biosynthetic pathway. Table S3 Primers for the clone of reannotated genes. Table S4 Summary of functional annotation results. Table S5 Distribution of transcriptional factors in the genomes of Camptotheca acuminata and Ophiorrhiza pumila. Table S6 Summary of comparative genome analysis results in DIMORPH. [file 43897_2025_225_MOESM1_ESM.docx]

**Supplementary Information**

Figure S1 Alignment of the newly generated raw transcriptome data of *Camptotheca acuminata* with *CacGene27004* and *CacGene08149*.


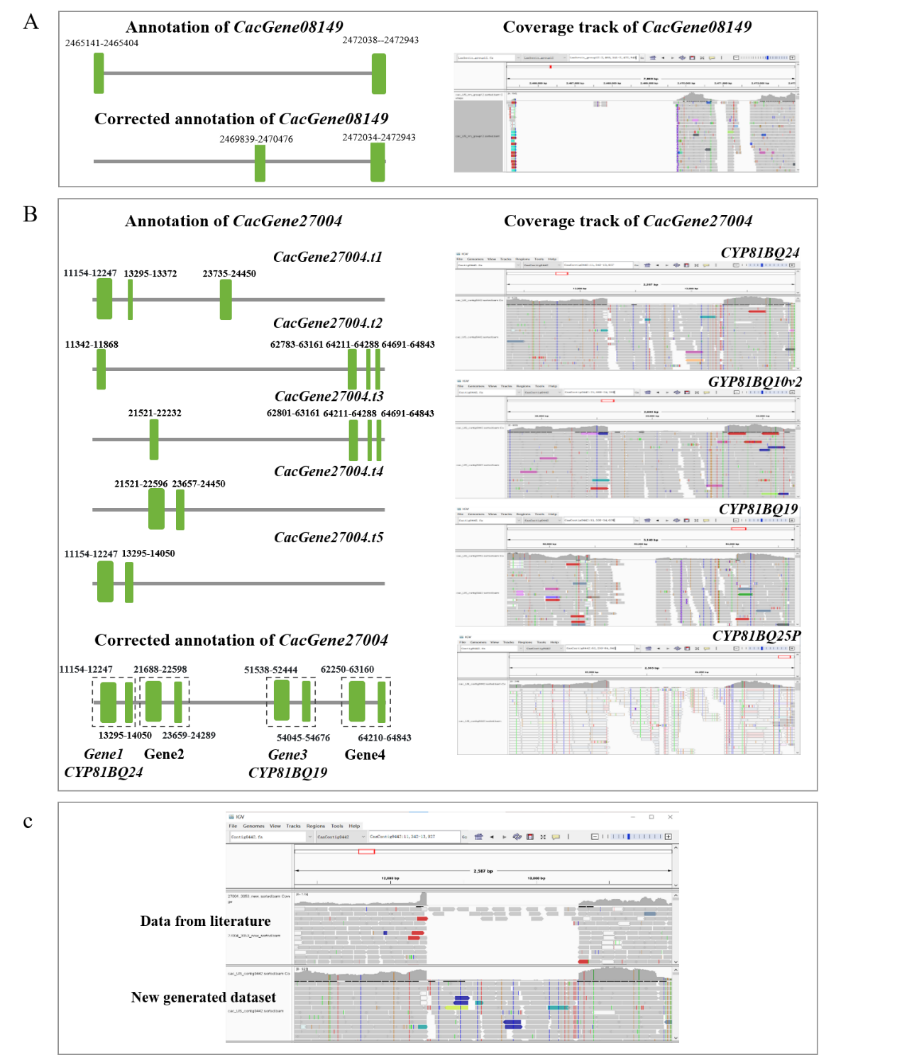


Figure S2 Phylogenetic relationship of CYP450s in the genome of *Camptotheca acuminata*.


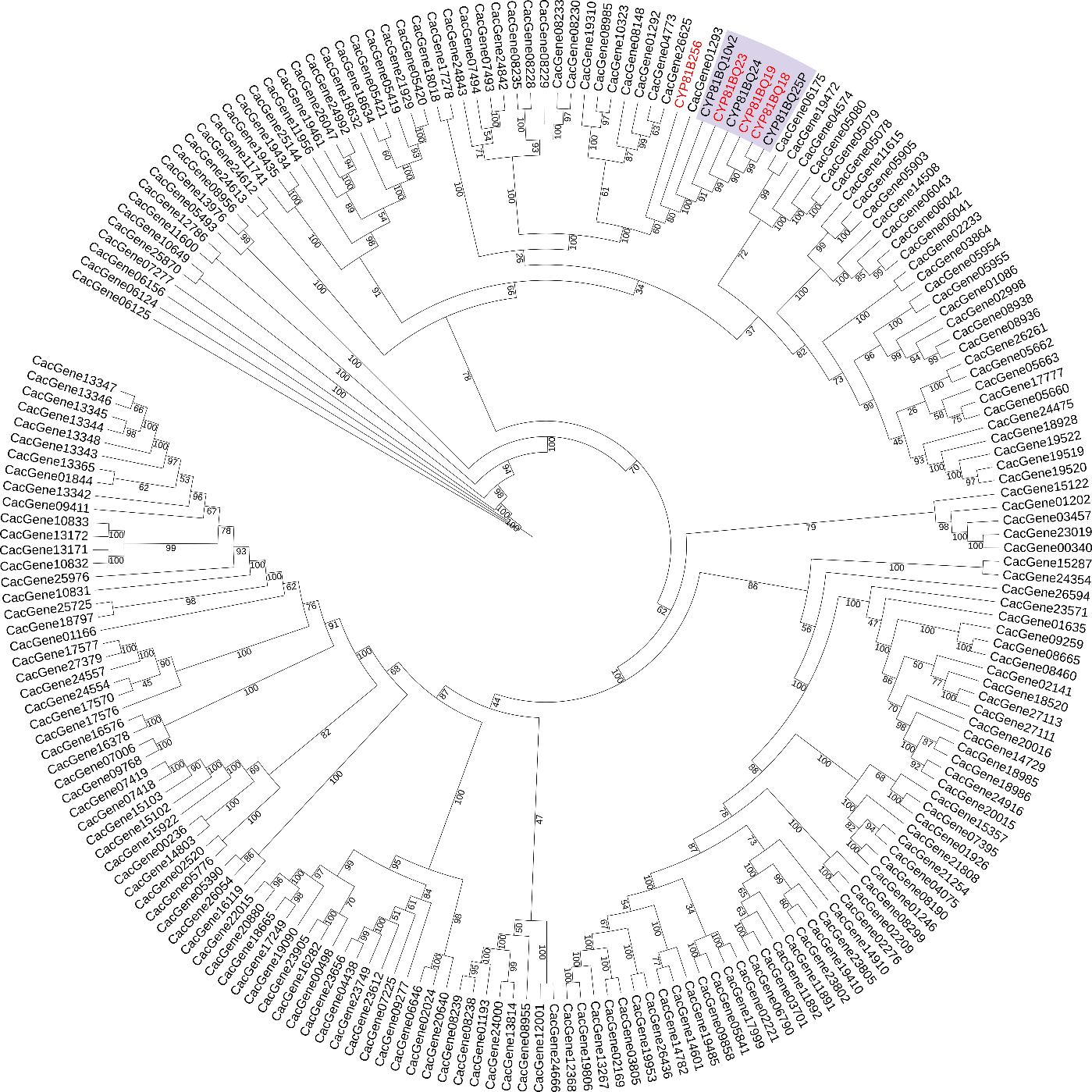


Figure S3 The MS spectra, ultraviolet absorption peak of the products of camptothecin hydroxylases. (A) Characteristic cleavages. (B) Ultraviolet absorption peak. (C) Extracted ion chromatograms from LC-MS analysis of *in vitro* reactions catalyzed by CYP81BQ24.


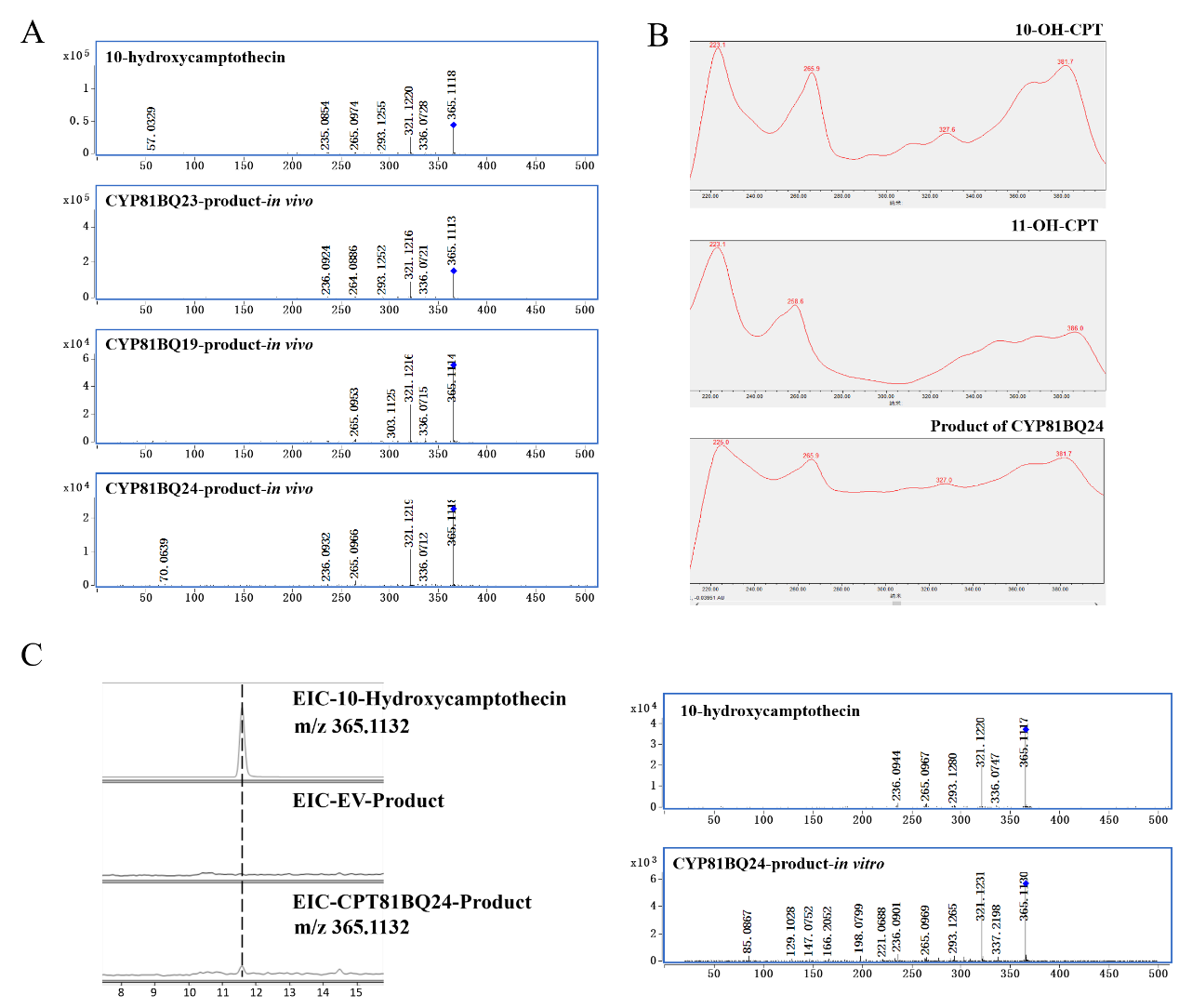


Figure S4 The comparison of catalytic activity of CPT 10-hydroxylases *in vivo.* * *P*<0.05; ** *P*<0.01, *** *P*<0.001; **** *P*<0.0001.

**
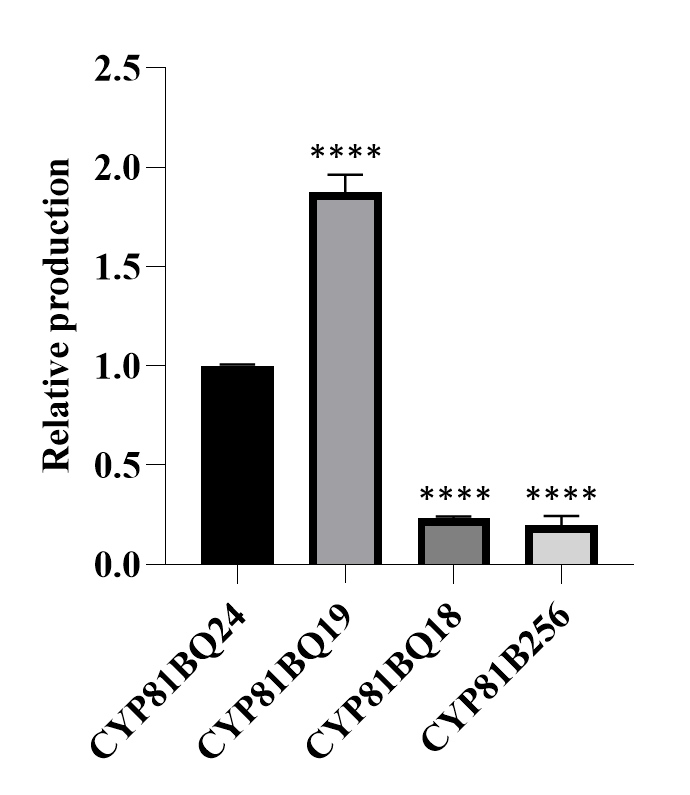
**

Figure S5 Sequence alignment of CYP81BQ24, CYP81BQ19 and CYP81BQ23 and identification of substrate recognition sites.


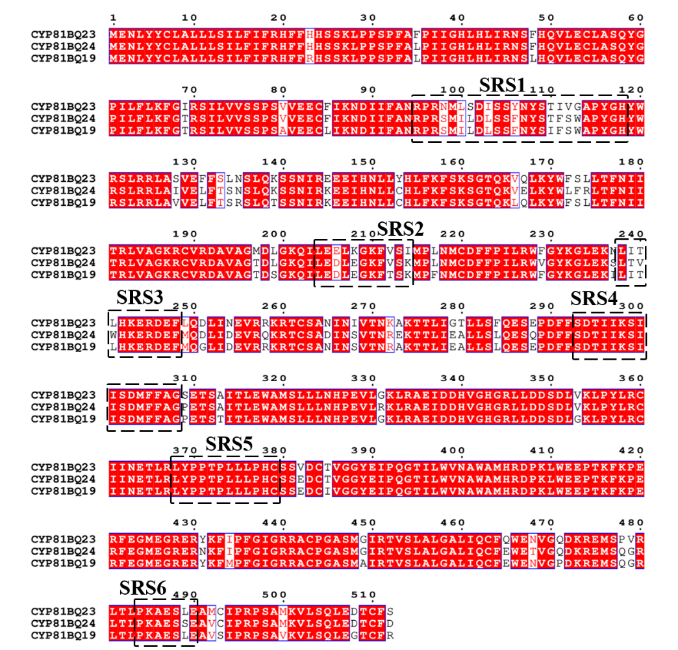


Figure S6 Sequence conservation analysis of CYP81BQ19 key differential sites in a wide range.


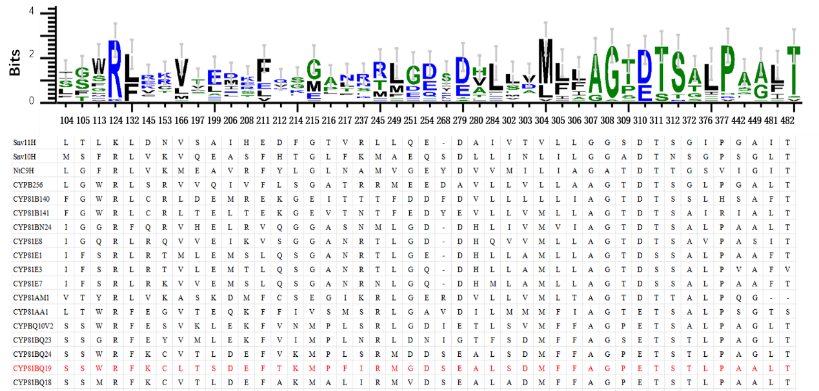


Figure S7 Molecular docking and substrate entrance of CYP81BQ19 and CYP81BQ19TM. (A) CYP81BQ19. (B) CYP81BQ19TM. The protein is shown as cartoon (light yellow). The substrate ligand is shown as green stick structures (red represents O atom, blue represents N atom, and green represents C atom), heme is shown as gray stick structures, the three mutation sites are shown as purple stick structures, the amino acids forming hydrogen bonds with the substrate are shown as red stick structures, yellow dashed lines represent hydrogen bonds, and green dashed lines represent the distance between the heme active center and the C-10 position of camptothecin.


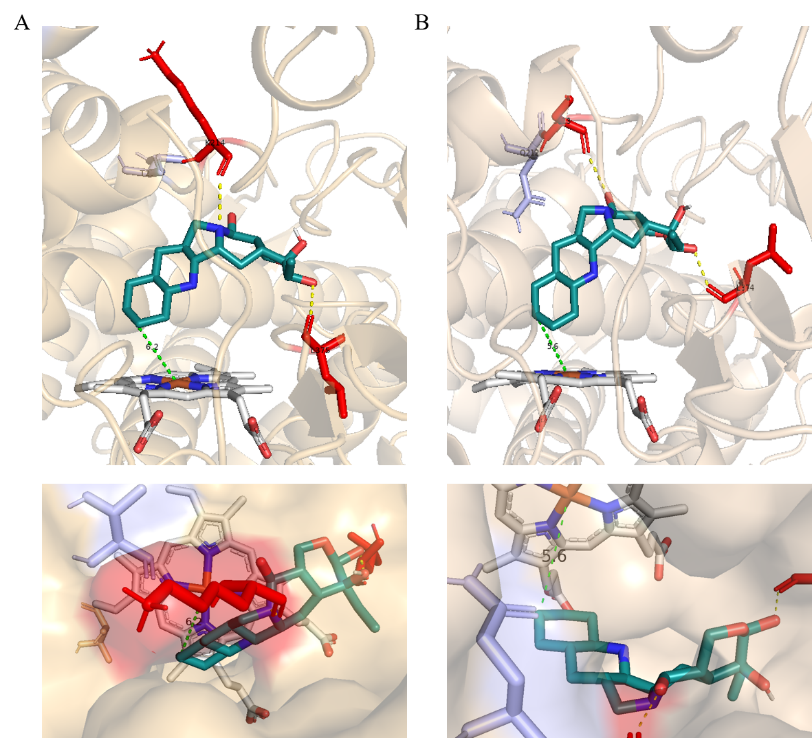


Table S1 RNA samples used in this study

| Species | Tissues | Samples | Origin |  |
| --- | --- | --- | --- | --- |
|  |  |  |  |  |
| *Camptotheca acuminata* | Low stem | 3 | This study |  |
|  | Mid stem | 3 |  |  |
|  | Up stem | 3 |  |  |
|  | Root | 3 |  |  |
|  | Young leaf | 3 |  |  |
|  | Mature leaf | 3 |  |  |
|  | Lower bark | 1 | (Kang et al., 2021) |  |
|  | Immature leaf | 1 |  |  |
|  | Callus | 1 |  |  |
|  | Root culture | 1 |  |  |
|  | Mature leaf | 2 |  |  |
|  | Immature flower | 1 |  |  |
|  | Immature fruit | 1 |  |  |
|  | Mature fruit | 1 |  |  |
|  | Trichomes | 1 |  |  |
|  | Seedling lateral roots | 1 |  |  |
|  | Seedling leaf | 1 |  |  |
|  | Seedling tap root | 1 |  |  |
|  | Seedling cotyledons | 1 |  |  |
|  | Seedling apical stem | 1 |  |  |
|  | Seedling basal stem | 1 |  |  |
|  | Whole seedlings | 1 |  |  |
|  | Upper bark | 1 |  |  |
|  | Entire root | 1 |  |  |
| *Ophiorrhiza pumila* | Fruit* | 2 | This study |  |
|  | Root | 3 |  |  |
|  | Stem  Leaf* | 3  0 |  |  |
| *Nothapodytes nimmoniana* | Root | 1 | (Rather et al., 2018) |  |
|  | Leaf | 1 |  |  |

Fruit*: A total of three fruit samples were collected, but the RNA extracted from one of the samples was of poor quality, and these data were removed during data processing.

Leaf*: A total of three samples were collected, but RNA extraction was unsuccessful, presumably due to the high polysaccharide content.

Table S2 MS/MS fragment profiles of key metabolites involved in the camptothecin biosynthetic pathway

| Compound | Chemical formula | Molecular formula | [M+H]^+^ (m/z) | MS/MS Fragments | Reference |
| --- | --- | --- | --- | --- | --- |
| Loganic acid |  | C_16_H_24_O_10_ | 394.1703 [M+NH4]+ | 359.1332,341.1227,323.1077,215.0910,197.0807,179.0701,151.0747,137.0581, 109.0654 | (Jin et al., 2019) |
|  |  |  | 377.145 | 359,215, 197, 179, 161, 151, 137, 133, 123, 109, 81 | (Sadre et al., 2016) |
|  |  |  |  |  |  |
| Loganin |  | C_17_H_26_O_10_ | 391.2599 | 371, 327, 283, 239, 195, 177, 133, 89 | (Yang et al., 2021) |
|  |  |  |  |  |  |
| Secologanin |  | C_17_H_24_O_10_ | 389.1459 | 227, 209, 195, 177, 165, 151, 149, 139, 109, 107 | (Yang et al., 2021) |
|  |  |  |  |  |  |
| Secologanic acid |  | C_16_H_22_O_10_ | 397.1009[M+Na]^+^ | 235.0573, 217.0468 | (Jin et al., 2019) |
|  |  |  | 375.129 | 213, 195, 177, 151, 125, 109, 107, 95, 79, 77 | (Sadre et al., 2016) |
|  |  |  |  |  |  |
| Tryptamine |  | C_10_H_12_N_2_ | 161.107 | 144.0788 | (Jin et al., 2019) |
|  |  |  | 161.1065 | 144 | (Sadre et al., 2016) |
| Strictosidine |  | C_27_H_34_N_2_O_9_ | 531.2356 | 355, 335, 283, 237, 163, 133, 118 | (Yang et al., 2021) |
|  |  |  |  |  |  |
| Strictosidinic acid |  | C_26_H_32_N_2_O_9_ | 517.2173 | 500.1907, 355.1648, 338.1383, 320.1279, 170.0960, 500.1909 | (Jin et al., 2019) |
|  |  |  | 517.2173 | 500.1909, 355.1648 | (Jin et al., 2019) |
|  |  |  | 517.219 | 500, 355, 338, 320, 269/268, 251, 194, 181/180, 170/168, 156, 151, 144, 125 | (Yang et al., 2021) |
|  |  | 3 isomers | 517.2198 | 500, 355, 338, 320, 269/268, 251, | (Sadre et al., 2016) |
|  |  |  |  | 194, 180/181, 168/170, 151, |  |
|  |  |  |  | 156, 144, 130, 125 |  |
| Strictosamide |  | C_26_H_30_N_2_O_8_ | 499.2065 | 337.1546, 267.1119 | (Jin et al., 2019) |
|  |  |  | 499.2081 | 337, 319, 267, 171, 144 | (Sadre et al., 2016) |
|  |  |  | 499.2083 | 337, 319, 267, 171, 144 | (Yang et al., 2021) |
|  |  |  |  |  |  |
| Strictosamide epoxide |  | C_26_H_30_N_2_O_9_ | 515.2016 | 353.1493, 335.1365, 283.1079 | (Jin et al., 2019) |
|  |  |  | 515.2025 | 353, 335, 309, 283, 265, 238, 209, 184, 144 | (Yang et al., 2021) |
|  |  | 3 isomers | 515.2021 | 353, 335, 309, 291, 283, 265, 263, 237, 209, 183, 184, 155, 144 | (Sadre et al., 2016) |
|  |  |  |  |  |  |
|  |  |  |  |  |  |
| Strictosamide diol |  | C_26_H_32_N_2_O_10_ | 533.2134 | 371, 353, 283, 265, 160, 142, 132 | (Yang et al., 2021) |
|  |  |  | 533.2183 | 371, 353, 283, 265, 185, 160, 142, 132 | (Sadre et al., 2016) |
|  |  |  |  |  |  |
| Strictosamide ketolactam |  | C_26_H_30_N_2_O_10_ | 531.2366 | 369, 351, 299, 281, 271, 253, 194, 176, 158 148, 130, 124, 106 | (Yang et al., 2021) |
|  |  |  |  |  |  |
| Pumiloside |  | C_26_H_28_N_2_O_9_ | 513.1861 | 351.1336, 333.1232, 315.1125, 281.0921 | (Jin et al., 2019) |
|  |  |  | 513.189 | 351, 333, 315, 305, 281, 235, 140 | (Sadre et al., 2016) |
|  |  |  | 513.1885 | 351, 333, 315, 305, 281, 263, 235, 140 | (Yang et al., 2021) |
|  |  |  |  |  |  |
| Deoxypumiloside |  | C_26_H_28_N_2_O_8_ | 497.1914 | 335.1388, 265.0973, 247.0868, 219.0914, 169.0761, 142.0654 | (Jin et al., 2019) |
|  |  |  | 497.1913 | 355.1384, 265.0970, 247.0868 | (Jin et al., 2019) |
|  |  |  | 497.193 | 335, 265, 247, 219, 183, 169, 142, 97 | (Sadre et al., 2016) |
|  |  |  | 497.1931 | 335, 265, 247, 219, 183, 169, 142 | (Yang et al., 2021) |
|  |  |  |  |  |  |
|  |  |  |  |  |  |
| Camptothecin |  | C_20_H_16_N_2_O_4_ | 349.1178 | 305.1285, 277.1324, 249.1017 | (Jin et al., 2019) |
|  |  |  | 349.1192 | 305, 277, 249, 219/220, 168 | (Yang et al., 2021) |
|  |  |  |  |  |  |
|  |  |  |  |  |  |
| Methoxycamptothecin |  | C_21_H_18_N_2_O_5_ | 379.1297 | 335, 325, 307, 292, 279, 264, 250, 235, 221, 211, 198, 156 | (Yang et al., 2021) |

Table S3 Primers for the clone of reannotated genes

| Gene | Primer (5’-3’) |
| --- | --- |
| *CYP81BQ24*-F | ATGGAGAACTTGTACTACTGC |
| *CYP81BQ24*-R | CTAATCGAAACAAGTGTCCTC |
| *CYP81BQ23*-F | ATGGAGAACTTGTACTACTGC |
| *CYP81BQ23*-R | CTAACTGAAACAAGTGTCTTC |
| *CYP81BQ19*-F | ATGGAGAACTTGTACTACTGC |
| *CYP81BQ19*-R | CTAACGGAAACAAGTGCCTTC |
| *CYP81BQ10v2*-F | ATGGAGAAGTTGTACTACTGCCTT |
| *CYP81BQ10v2*-R | CTAAATAAAGGAAGTGTCTTCAAGCTG |
| *CYP81BQ25P*-F | ATGGAGAACTTGTACTACTGCCTT |
| *CYP81BQ25P*-R | CTAACTGAAACAAGTGTCTTCTGGCTG |

Table S4 Summary of functional annotation results

|  | *Camptotheca acuminata* (2017) | *Camptotheca acuminata* （2021） | *Ophiorrhiza pumila* （2021） |
| --- | --- | --- | --- |
| Gene | 31 825 | 27 940 | 32 374 |
| NR | 28 835 | 26 052 | 29 391 |
| Swiss-Prot | 22 323 | 20 600 | 18 774 |
| GO | 15 010 | 13 793 | 12 620 |
| KEGG | 13 415 | 12 524 | 9 991 |
| Pfam | 23 213 | 21 243 | 20 571 |

Table S5 Distribution of transcriptional factors in the genomes of *Camptotheca acuminata* and *Ophiorrhiza pumila*

|  | *Camptotheca acuminata* （2017） | *Camptotheca acuminata* （2021） | *Ophiorrhiza pumila* （2021） |
| --- | --- | --- | --- |
| MYB | 173 | 186 | 88 |
| AP2/ERF-ERF | 177 | 152 | 88 |
| bHLH | 149 | 152 | 95 |
| C2H2 | 149 | 144 | 89 |
| WRKY | 97 | 100 | 49 |
| NAC | 117 | 92 | 90 |
| C3H | 74 | 75 | 44 |
| GRAS | 87 | 75 | 58 |
| MYB-related | 79 | 69 | 40 |
| bZIP | 68 | 64 | 44 |
| GARP-G2-like | 57 | 52 | 36 |
| HB-HD-ZIP | 51 | 51 | 27 |
| LOB | 54 | 50 | 41 |
| B3 | 69 | 46 | 205 |
| GNAT | 37 | 32 | 27 |
| Other family | 78 | 79 | 76 |
| Total family | 93 | 94 | 91 |
| Total genes | 2616 | 2478 | 1770 |

Table S6 Summary of comparative genome analysis results in DIMORPH

|  | Synteny block | Collinear gene pair |
| --- | --- | --- |
| *C. acuminata* (2017) vs *C. acuminata* (2021) | 1283 | 46211 |
| *C. acuminata* (2017) vs *O. pumila* (Published in 2021) | 850 | 29658 |
| C. acuminata (2021) vs O. pumila (Published in 2021) | 618 | 28845 |

Reference

**Jin, Z., Wan, R., Yan, R., Su, Y., Huang, H., Zi, L., and Yu, F.** (2019). Microwave-Assisted Extraction of Multiple Trace Levels of Intermediate Metabolites for Camptothecin Biosynthesis in Camptotheca acuminata and Their Simultaneous Determination by HPLC-LTQ-Orbitrap-MS/MS and HPLC-TSQ-MS. Molecules (Basel, Switzerland) **24,** 815.

**Kang, M., Fu, R., Zhang, P., Lou, S., Yang, X., Chen, Y., Ma, T., Zhang, Y., Xi, Z., and Liu, J.** (2021). A chromosome-level Camptotheca acuminata genome assembly provides insights into the evolutionary origin of camptothecin biosynthesis. Nature communications **12,** 3531.

**Rather, G., Sharma, A., Pandith, S., Kaul, V., Nandi, U., Misra, P., and Lattoo, S.K.** (2018). De novo transcriptome analyses reveals putative pathway genes involved in biosynthesis and regulation of camptothecin in Nothapodytes nimmoniana (Graham) Mabb. Plant molecular biology **96,** 197-215.

**Sadre, R., Magallanes-Lundback, M., Pradhan, S., Salim, V., Mesberg, A., Jones, A.D., and DellaPenna, D.** (2016). Metabolite Diversity in Alkaloid Biosynthesis: A Multilane (Diastereomer) Highway for Camptothecin Synthesis in Camptotheca acuminata. The Plant cell **28,** 1926-1944.

**Yang, M., Wang, Q., Liu, Y., Hao, X., Wang, C., Liang, Y., Chen, J., Xiao, Y., and Kai, G.** (2021). Divergent camptothecin biosynthetic pathway in Ophiorrhiza pumila. BMC biology **19,** 122.
